# Focal Local Field Potential Signature of the Single-Axon Monosynaptic Thalamocortical Connection

**DOI:** 10.1523/JNEUROSCI.2715-16.2017

**Published:** 2017-05-17

**Authors:** Espen Hagen, Janne C. Fossum, Klas H. Pettersen, Jose-Manuel Alonso, Harvey A. Swadlow, Gaute T. Einevoll

**Affiliations:** ^1^Faculty of Science and Technology, Norwegian University of Life Sciences, Aas, Norway,; ^2^Institute of Neuroscience and Medicine (INM-6) and Institute for Advanced Simulation (IAS-6) and Jülich Aachen Research Alliance - Brain Institute I, Jülich Research Centre, 52425 Jülich, Germany,; ^3^Department of Physics and; ^4^Letten Centre and GliaLab, Institute of Basic Medical Sciences, University of Oslo, 0316 Oslo, Norway,; ^5^Department of Biological Sciences, State University of New York College of Optometry, New York, New York 10036, and; ^6^Department of Psychology, University of Connecticut, Storrs, Connecticut 06269

**Keywords:** layer 4, local field potential, modeling, monosynaptic, sensory cortex, thalamocortical

## Abstract

A resurgence has taken place in recent years in the use of the extracellularly recorded local field potential (LFP) to investigate neural network activity. To probe monosynaptic thalamic activation of cortical postsynaptic target cells, so called spike-trigger-averaged LFP (stLFP) signatures have been measured. In these experiments, the cortical LFP is measured by multielectrodes covering several cortical lamina and averaged on spontaneous spikes of thalamocortical (TC) cells. Using a well established forward-modeling scheme, we investigated the biophysical origin of this stLFP signature with simultaneous synaptic activation of cortical layer-4 neurons, mimicking the effect of a single afferent spike from a single TC neuron. Constrained by previously measured intracellular responses of the main postsynaptic target cell types and with biologically plausible assumptions regarding the spatial distribution of thalamic synaptic inputs into layer 4, the model predicted characteristic contributions to monosynaptic stLFP signatures both for the regular-spiking (RS) excitatory neurons and the fast-spiking (FS) inhibitory interneurons. In particular, the FS cells generated stLFP signatures of shorter temporal duration than the RS cells. Added together, a sum of the stLFP signatures of these two principal synaptic targets of TC cells were observed to resemble experimentally measured stLFP signatures. Outside the volume targeted by TC afferents, the resulting postsynaptic LFP signals were found to be sharply attenuated. This implies that such stLFP signatures provide a very local measure of TC synaptic activation, and that newly developed inverse current-source density (CSD)-estimation methods are needed for precise assessment of the underlying spatiotemporal CSD profiles.

**SIGNIFICANCE STATEMENT** Despite its long history and prevalent use, the proper interpretation of the extracellularly recorded local field potential (LFP) is still not fully established. Here we investigate by biophysical modeling the origin of the focal LFP signature of the single-axon monosynaptic thalamocortical connection as measured by spike-trigger-averaging of cortical LFPs on spontaneous spikes of thalamocortical neurons. We find that this LFP signature is well accounted for by a model assuming thalamic projections to two cortical layer-4 cell populations: one excitatory (putatively regular-spiking cells) and one inhibitory (putatively fast-spiking cells). The LFP signature is observed to decay sharply outside the cortical region receiving the thalamocortical projection, implying that it indeed provides a very local measure of thalamocortical synaptic activation.

## Introduction

**Figure 1.**
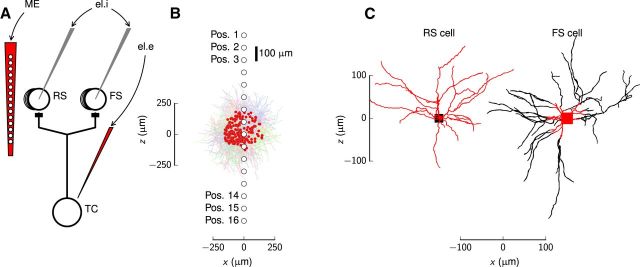
Illustration of the TC connection to the cortex, postsynaptic model populations, and reconstructed morphologies with corresponding synapse sites. ***A***, Schematic view of the monosynaptic TC projection to layer 4 and corresponding measurements of responses for an axon of a single TC cell connecting monosynaptically to RS and FS cells in layer 4. ME, Extracellular multicontact electrode recording the LFP through the cortex; el.i, whole-cell patch-clamp electrodes that record intracellular responses in terms of EPSPs and corresponding currents; el.e, extracellular recording electrode in the topographically aligned thalamic region that detects or initiates spiking activity in the TC cell. ***B***, Postsynaptic model populations shown with 40 reconstructed stellate cells (RS cells) and 10 reconstructed interneurons (FS cells). Locations of synaptic inputs from the TC cell are denoted by red dots, all within a radius of 165 μm from the center of the synaptic projection pattern (compare Eq. 4). The locations of ME contact points penetrating the population are denoted by circles labeled Pos. 1–16. ***C***, Reconstructed morphologies of the RS principal neuron and FS interneuron employed in the model populations. Putative locations of synaptic inputs of TC afferents onto the RS cell and FS cell are marked in red, i.e., dendritic compartments of the RS cell and within 50 μm of, and including, the soma for the FS cell.

The use of the extracellularly recorded local field potential (LFP) in probing cortical neural activity has seen a revival in the last decade (Buzsáki et al., 2012; Einevoll et al., 2013). This signal, which results from transmembrane currents in thousands of cells in the neural tissue surrounding the recording contact, is generally difficult to interpret, and a detailed mathematical analysis is needed to relate the signal to the underlying activity of neurons (Einevoll et al., 2013). Here we model and investigate one type of such recordings, i.e., the spike-trigger-averaged LFP (stLFP*)* of monosynaptic connections from single thalamocortical (TC) neurons impinging onto neuronal populations in cortical layer 4 (Swadlow et al., 2002), as illustrated schematically in Figure 1*A*.

Layer 4 is considered the major gateway into the cortex of sensory information relayed by the thalamus (Bruno and Sakmann, 2006). Axons of TC neurons form synapses with both excitatory regular-spiking (RS) cells and inhibitory fast-spiking (FS) interneurons (Gabernet et al., 2005; Bruno and Sakmann, 2006; Inoue and Imoto, 2006; Cruikshank et al., 2007; Hull et al., 2009; Oberlaender et al., 2012; Schoonover et al., 2014). In studies of this TC connection, intracellular responses in cortical neurons have been assessed in whole-cell patch-clamp recordings as EPSCs or EPSPs (Beierlein et al., 2003; Bruno and Sakmann, 2006; Cruikshank et al., 2007; Hull et al., 2009). An alternative approach is to record extracellular stLFP signatures of the TC activation of the postsynaptic target cells by simultaneous spike recordings in thalamus and multielectrode recordings in the cortex. For example, in Swadlow et al. (2002) cortical responses in the rabbit somatosensory cortex from TC neurons in the topographically aligned ventrobasal thalamus were assessed in terms of such spatiotemporal stLFP signatures, and similar methodology has been applied in other species and other cortical regions (Bereshpolova et al., 2006; Jin et al., 2008, 2011; Stoelzel et al., 2008, 2009).

In this study we investigated the biophysical origin of these stLFP signatures with a mathematical forward-modeling scheme (Einevoll et al., 2013). Specifically, we computed such signatures in layer-4 model populations incorporating biophysically detailed RS-cell and FS-cell models constrained by available anatomical (Freund et al., 1985; da Costa and Martin, 2009; Oberlaender et al., 2012) and physiological (Beierlein et al., 2003; Hull et al., 2009) data. We found that (1) with model parameters tuned to be in accordance with previous experimental recordings of EPSPs and EPSCs, and (2) with excitatory synaptic inputs assumed located on dendritic sections of RS cells, and on somas and soma-proximal dendrites of FS cells, the model predicts stLFP signatures in agreement with previously published experiments (Swadlow et al., 2002; Stoelzel et al., 2008). The model findings further suggested that the RS-cell and FS-cell populations have distinct contributions to the short-latency part of the postsynaptic signature with the FS-cell contribution exhibiting a sharper temporal profile. Importantly, the model predicted the magnitude of the stLFP signals to rapidly decay in the lateral directions outside the synaptically activated cortical regions, demonstrating that the measurement of such signals indeed provides a very local measure of neural activity as observed experimentally (Swadlow and Gusev, 2000).

Experimental stLFP signatures have previously been analyzed using current-source density (CSD) analysis to identify the relative strength and locus of synaptic input of TC projections onto cortical populations. We also used our model results, where the “ground truth” is known, to test the accuracy of various methods for estimation of the CSD (Pettersen et al., 2008). While the traditional CSD method, based on taking the “double spatial derivative” (Freeman and Nicholson, 1975; Mitzdorf, 1985), failed as expected to predict an accurate spatial profile due to the small lateral extension of the present monosynaptic neural activation, appropriately chosen inverse CSD (iCSD) methods (Pettersen et al., 2006) were found to give more accurate CSD estimates.

## Materials and Methods

### 

#### Neural population model

##### Multicompartmental neuron models.

The layer-4 model population consisted of two types of neurons: RS-cell neurons and FS-cell neurons (Beierlein et al., 2003; Hull et al., 2009). Reconstructed morphologies, presumably representative for these two types were obtained from http://www.neuromorpho.org (RRID:SCR_002145; Ascoli et al., 2007). We chose a spiny stellate-cell morphology for the RS-cell neuron (NeuroMorpho.org ID: NMO_00300) and a large basket-cell morphology for the FS-cell neuron (NeuroMorpho.org ID: NMO_00299), both from Wang et al. (2002; Fig. 1*C*). These two morphologies were selected as they had proper 3D representations and seemingly few missing dendritic branches. The axonal sections were removed.

Spine areas onto dendritic sections of RS cells were compensated for, as reported by Major et al. (1994) and Mainen and Sejnowski (1996), using a factor *F* = (*LA*_spine_ρ_spine_ + *A*_sec_)/*A*_sec_, where *L* is the unperturbed section length; *A*_spine_, the spine area, is 0.83 μm^2^ (Harris and Stevens, 1989); ρ_spine_, spine density, is 1 μm^−1^ (Mainen and Sejnowski, 1996), and *A*_sec_ is the unperturbed section membrane surface area. Then, each section length *L* and diameter *d* were multiplied by *F*^2/3^ and *F*^1/3^, respectively, before compartmentalization.

In the discretized cable models (Holmes, 2009) of each neuron, the dendrites were assumed to be passive, and the number of compartments for each individual dendritic section was set according to the d_lambda rule (Hines and Carnevale, 2001) with parameters *d* = 0.1 and *f* = 1000 Hz. The electrotonically compact soma sections were, however, segmented into 11 compartments. The resulting total number of compartments were 575 for the RS cell and 827 for the FS cell. The number of compartments per dendritic section is always an odd number, and the distribution of transmembrane currents along each cylindrical compartment is assumed to be homogeneous.

We used conductance-based synapse models, where the synaptic currents *I*_syn_ were modeled as follows (Eq. 1):

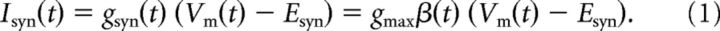
 Here *V*_m_(*t*) is the membrane potential, *E*_syn_ is the reversal potential of synaptic conductance, *g*_syn_(*t*) is the synapse conductance, and *g*_max_ is the maximal conductance. The function β(*t*) determined the time course of the conductance modeled as a difference of two exponentials, specified by rise (τ_rise_), decay (τ_decay_), and delay (τ_delay_) time constants (Roth and van Rossum, 2009). The equation (Eq. 2) is as follows:

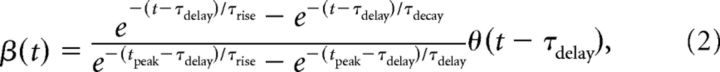
 where θ(*t*) is the unit step function (θ(*t* ≥ 0) = 1, θ(*t* < 0) = 0), and *t*_peak_ is the time of maximum conductance given by the following (Eq. 3):

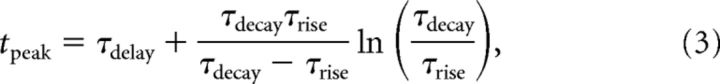
 Note that β(*t*_peak_) = 1, so that the maximal conductance is indeed *g*_max_ in Equation 1.

Neuron and synapse parameters used for RS and FS cells are summarized in Table 1.

**Table 1.** Summary of default simulation parameters for the RS-cell and FS-cell populations

Parameter	Description	RS cell	FS cell	Unit
*n*_pop_	Population size	4000	1000	—
*r*	Population radius	500	500	μm
*h*	Population thickness	500	500	μm
*n̄*_syn_	Imposed synapse count	7*^d^*	15*^e^*	—
	Synapse location	Dendrites	Soma/proximal	—
*W_i_*	Projection	Spherical	Spherical	—
*r*_syn_	Synapse projection radius	165	165	μm
μ*_z_*	Synapse projection offset	−35	0	μm
Morphology	NeuroMorpho.Org ID	NMO_00300	NMO_00299	—
*C*_m_	Specific membrane capacitance	0.9*^b^*	0.9*^b^*	μF · cm^−2^
*R*_m_	Specific membrane resistance	11,250	5625	Ω · cm^2^
*R*_a_	Specific axial resistance	150*^c^*	150*^c^*	Ω · cm
*V*_rest_	Resting membrane potential	−66*^a^*	−64*^a^*	mV
*n*_seg_	Segment count	575	827	—
*R*_DC_	DC input resistance	129.7	37.7	MΩ
τ_rise_	Synaptic rise time constant	0.20	0.05	ms
τ_decay_	Synaptic decay time constant	2.00	0.20	ms
τ_delay_	Synaptic activation delay	1.4/0*	1.4/0*	ms
*g*_max_	Synaptic conductance	0.40	1.75	nS
*E*_syn_	Synaptic reversal potential	0	0	mV

Model parameters. Upper set of parameters corresponds to the populations and synapse projections; middle set gives specific neuron parameters; bottom set gives parameters for synapse models. Some parameters were taken directly from the literature: *^a^p*Beierlein et al., 2003;

*^b^*Gentet et al., 2000;

*^c^*Mainen and Sejnowski, 1996;

*^d^*Gil et al., 1999,

*^e^*Gabernet et al., 2005. *, Synaptic activation at τ_delay_ = 0 ms was used for Figures 5–9.

##### Cortical cell populations.

Model populations of RS and FS cells were constructed by randomly assigning soma positions of the neurons with homogeneous density inside predefined cylindrical volumes. Unless otherwise specified, cylinders with height *h* and radius *r* of 500 μm were used. This assumed layer thickness *h* is consistent with data reported by others, such as Oberlaender et al. (2012), who found a thickness of layer 4 in rat barrel cortex of ∼480 μm. The centers of the populations were defined to be the origin (*x* = *y* = *z* = 0). A random rotation around all three rotation axes were applied for each neuron. The default numbers of neurons used for the simulations were 4000 RS cells and 1000 FS cells (compare Table 1).

##### Placement of TC synapses onto populations.

From the literature it is known that synaptic projections from thalamic cells onto FS cells are primarily located on somas or dendrites proximal to soma (White et al., 1984; Keller and White, 1987; Staiger et al., 1996; Ahmed et al., 1997; Porter et al., 2001; Bagnall et al., 2011), while thalamic projections on RS cells are predominantly located on spines in the dendritic arbors (Keller and White, 1987; Ahmed et al., 1994; Banitt et al., 2007; da Costa and Martin, 2011; da Costa, 2013). Here we thus assumed that synapses onto RS cells could connect on dendrites only, while synapses onto FS cells could connect on somatic and proximal dendritic compartments, that is, only onto dendritic compartments for which each center was less than a radial distance of 50 μm from the center of the soma. These synaptic locations are illustrated on the corresponding RS-cell and FS-cell reconstructions in Figure 1*C*.

After each neuron was assigned a position within its population, the synaptic placements of thalamic axons onto RS and FS cells were determined by first defining a geometrical synaptic connection rule. This rule depended on the desired 3D shape of the synaptic projection patterns, and three different rules were considered, each described by a function *W_i_*.

The first rule considered is the spherical projection pattern. The spherically shaped synaptic target region with radius *r*_syn_ with sharp boundaries where the spatial function *W_i_*, evaluated for each compartment of each neuron in the population, is given by the following (Eq. 4):

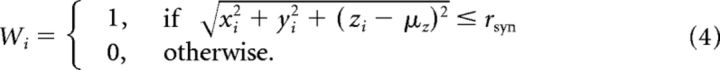
 The variables *x_i_*, *y_i_*, *z_i_* are midpoint positions of the compartments, and μ*_z_* is the vertical offset of the synaptic projection.

The second rule considered is the cylindrical projection pattern. These cylindrically shaped synaptic target regions with sharp boundaries are determined using spatial functions *W_i_* on the following form (Eq. 5):

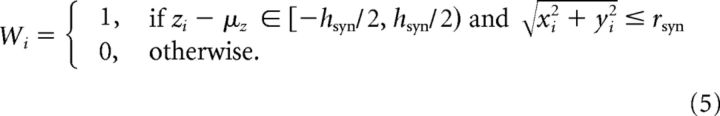
 Here *h*_syn_ is the height of the synaptic target cylinder and *r*_syn_ its radius.

The third rule considered is the Gaussian projection pattern. This describes the synaptic target region as a Gaussian distribution with SDs (σ*_x_*, σ*_y_*, σ*_z_*) as follows (Eq. 6):

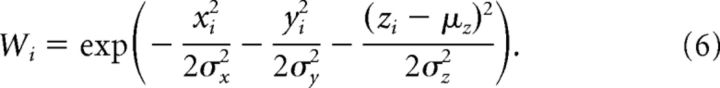
 We did not fix the number of synapses from each thalamic axon onto each neuron in the postsynaptic population. Rather, this number was determined in a stochastic procedure, introducing an auxiliary variable *p_i_*_,_*_A_* defined as follows:

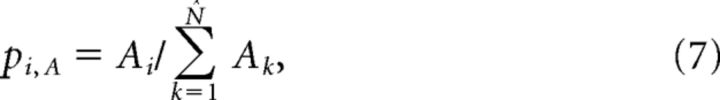
 describing the normalized probability for synaptic placement on a given compartment *i* with area *A_i_* of each cell. *A_k_* denotes membrane surface areas of each of *N̂* putative postsynaptic compartments, i.e., effectively all dendritic compartments for the RS cells and both somatic and dendritic compartments within 50 μm of the soma center for FS cells (see above).

Further, to position synapses at random, yet with a desired spatial profile (specified by the functions *W_i_*), while simultaneously taking into account the normalized probability of synaptic placement per compartment surface area (i.e., *p_i_*_,_*_A_*), we used the following algorithm: (1) a double for-loop iteratively ranged over the *N* compartments of the neuron and a random integer *n*_syn_ from a Poisson process with expectation *n̄*_syn_; (2) in every iteration the product *p_i_*_,_*_A_W_i_* was evaluated and a random number *X* drawn on the interval [0, 1); and (3) when the condition *X* < *p_i_*_,_*_A_W_i_* was true, a synapse was assigned to the corresponding compartment *i*.

With this procedure, cells with all dendrites placed within the target spatial profile (for the sharp-boundary spherical and cylindrical projection patterns, i.e., the first and second rules considered above) received approximately *n̄*_syn_ synaptic inputs, while other cells with stray sections in *W_i_*, typically received <*n̄*_syn_, or zero, synaptic inputs. Remaining cells received no synaptic input.

Following estimates from the literature, we imposed an average number of *n̄*_syn_ = 7 synaptic connections from a single thalamic neuron onto a single RS cell with all its dendrites placed within the synaptic target region (Gil et al., 1999; Bruno and Sakmann, 2006). For the corresponding projection onto an FS cell we used *n̄*_syn_ = 15 synaptic connections (Gabernet et al., 2005; Cruikshank et al., 2007; Hull et al., 2009; Bagnall et al., 2011).

##### Choice of model parameters.

The default model parameters, all listed in Table 1, were set by a mixed procedure. Some, like the resting membrane potential *V*_rest_ of the RS and FS cells, were taken directly from the literature (Table 1, numbers with letters in superscript). Others, in particular parameters describing the synapses, were chosen to give model results in qualitative agreement with *in vitro* slice recordings from similar cells in rodents (Beierlein et al., 2003; Hull et al., 2009). To illustrate the resulting agreement with experiments, Table 2 shows the parameters found when fitting of a double-exponential function of the type in Equation 2 to (1) experimental EPSPs extracted from Figure 10 in Beierlein et al. (2003) and (2) quantal EPSCs (presumably individual quanta of neurotransmitter from individual thalamic afferents) from Figure 3 in Hull et al. (2009), who also report monosynaptic EPSC amplitudes in RS-cell EPSCs of 56 ± 9 pA and in FS cells of 229 ± 57 pA. While we focused on these two studies when tuning the parameters, the RS-cell and FS-cell responses appear similar to those observed in other *in vitro* studies. Cruikshank et al. (2007), for example, also report significantly higher EPSC and EPSP amplitudes for thalamic connections on FS cells compared with RS cells, i.e., EPSCs of ∼250 and ∼30 pA, respectively. In terms of EPSP amplitudes, Bruno and Sakmann (2006) reported *in vivo* EPSP amplitudes from thalamic input into RS-like cells in rat that are lower by a factor of ∼2 compared with those obtained *in vitro*. However, this was possibly due to persistent synapse depression from spontaneous activity in thalamic neurons in their experiments. Experimental findings also indicate that FS cells have shorter time constants for EPSPs and EPSCs of thalamic input when compared with RS cells, which not only reflect the dynamics of the synapses, but also the typically shorter membrane time constants and smaller input resistance of layer-4 FS cells compared with layer-4 RS cells (for *in vivo* measurements in cat visual cortex, see Cardin et al. 2007; for *in vitro* measurements in slices of mouse somatosensory barrel cortex, see Hull et al., 2009; for *in vitro* preparations of rat barrel cortex, see Beierlein et al., 2003). The resultant DC input resistances of our RS-cell and FS-cell morphologies were 129.7 and 37.7 MΩ, respectively, values close to those reported by Beierlein et al. (2003).

**Table 2.** Summary of EPSPs and quantal EPSCs extracted from experimental data

Parameter	*V*_EPSP_ (RS)	*V*_EPSP_ (FS)	*i*_EPSC_ (RS)	*i*_EPSC_ (FS)
τ_delay_ (ms)	1.5	1.5	1.5	1.5
τ_rise_ (ms)	1.321	0.371	0.444	0.0892
τ_decay_ (ms)	11.796	5.491	2.510	0.5349
ω (amplitude)	2.4 mV	4.1 mV	7.2 pA	19.1 pA
*R*^2^	1.000	0.998	0.991	0.989

Time constants τ and amplitudes ω found from fits of two-exponential functions similar to Equation 2 and to data reported by Beierlein et al. (2003) and Hull et al. (2009).

To compare the predictions from our model to these experimental data, we (1) computed corresponding somatic EPSPs and somatic EPSCs (soma clamped at resting potentials for the latter) for every postsynaptic RS and FS cell in the populations using the default model parameters in Table 1. We then (2) computed the average EPSPs and EPSCs across the postsynaptic populations, and (3) fitted the two-exponential function in Equation 2 to these simulation results. The results are shown in Table 3 for the RS-cell and FS-cell populations, respectively, illustrating a qualitatively good agreement with the “two-exponential” parameters in Table 2 fitted to experimental data, primarily in terms of temporal envelopes, but EPSP amplitudes were also in reasonable agreement with the experimental findings.

**Table 3.** Averaged model synapse currents, EPSP responses, and EPSC responses

Parameter	ı̄_syn_	ı̄_EPSC_	*V̄*_EPSP_	Unit
τ_delay_	1.428	1.448	1.621	ms
τ_rise_	0.180	0.666	3.850	ms
τ_decay_	2.004	3.346	3.865	ms
Amplitude	−25.2 pA	−34.1 pA	1.043 mV	
*R*^2^	1.000	1.000	1.000	—
τ_delay_	1.426	1.428	1.463	ms
τ_rise_	0.044	0.054	0.116	ms
τ_decay_	0.202	0.257	2.017	ms
Amplitude	−106.4 pA	−784.1 pA	3.822 mV	
*R*^2^	1.000	0.999	0.993	—

Time constants and corresponding amplitudes found from fitting two-exponential functions (Eq. 2) to averaged RS-cell (top) and FS-cell (bottom) postsynaptic responses, here inferred as mean synapse current, EPSC, and EPSP.

To quantify goodness of fit between each signal *y* (averaged synapse currents, PSPs, PSCs) and best-fit *ŷ*, we calculated coefficients of determination (*R*^2^ values) defined as follows (Eq. 8):

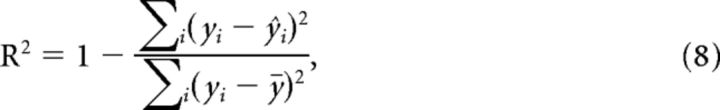
 where *ȳ* is the mean of the signal.

#### Calculations of extracellular potentials

Extracellular potentials are, according to the well established and presently used volume conductor theory, generated by transmembrane currents (Nunez and Srinivasan, 2006; Einevoll et al., 2013). Here, the extracellular medium is modeled as a smooth 3D continuum with neuronal transmembrane currents representing volume current sources. The fundamental formula relating neural activity in an infinite volume conductor to the generation of the LFP φ(*r*, *t*) at a (virtual) point electrode positioned at *r* is given by the following (Eq. 9; Holt and Koch, 1999; Pettersen et al., 2012; Lindén et al., 2013):

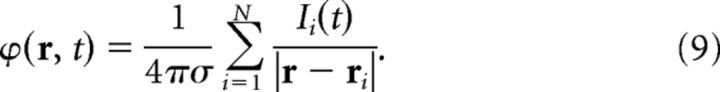
 Here, *I_i_*(*t*) denotes the transmembrane current (including the capacitive current) in a neural compartment *i* localized in a point positioned at *r_i_*. The extracellular conductivity, here assumed real (ohmic), isotropic (same in all directions), and homogeneous (same at all positions), is denoted by the scalar σ. The transmembrane compartment currents *I_i_*(*t*) were found by multicompartmental modeling using the simulation tool NEURON (Carnevale and Hines, 2006). In the numerical calculations of the LFP, facilitated by the Python package LFPy (Lindén et al., 2013), the line-source method resulting from integration of Equation 9 over linear segments was used (Holt and Koch, 1999). To avoid unphysical singularities that may result from dendritic segments positioned on top of point electrodes in the simulations, a minimum perpendicular distance between the line source and the point electrode position equal to the compartment radius was assumed. A scalar extracellular conductivity of σ = 0.3 S · m^−1^ was used (Hämäläinen et al., 1993; Goto et al. 2010).

LFPs generated by the model population were calculated in locations corresponding to electrode contacts of the laminar (linear) electrode used by Swadlow et al. (2002), Stoelzel et al. (2008), and Jin et al. (2008, 2011) with 16 contacts with an equidistance of 100 μm, inserted perpendicular to the cortical surface. In our simulations, electrode contact (channel) no. 9 was (unless otherwise noted) set to correspond to the vertical center of the postsynaptic populations, i.e., the origin. In most simulations, the electrode axis penetrated perpendicularly to the lateral center of the populations, but off-center situations were also considered. The finite size of the electrode contacts were taken into account by means of the so-called disc-electrode approximation (Lindén et al., 2013; Ness et al., 2015), i.e., by averaging the calculated potential across the surface of a flat, circular electrode contact of radius 15 μm [similar to the electrode contact sizes used by Swadlow et al. (2002), Stoelzel et al. (2008), and Jin et al. (2008, 2011)]. The normal vectors of the uninsulated electrode contact point surfaces were assumed perpendicular to the axis of the laminar electrode, and the returned potentials were obtained by averaging the potentials across 100 points drawn with uniform probability on each electrode's surface.

All simulations were executed using a temporal resolution of Δ*t* = 0.03125 ms (*f* = 32 kHz) with total simulation durations of 6 ms.

#### CSD

##### CSD estimation.

The CSD (i.e., the net current density entering or leaving the extracellular medium at a particular spatial position) was estimated from the LFP by means of two methods. In the traditional or standard CSD method (tCSD), the lateral variation of the LFP (and CSD) is implicitly assumed negligible so that the depth-resolved CSD can be estimated by the one-dimensional “double spatial derivative” of the LFP in the *z* direction (Freeman and Nicholson, 1975). Here the method of Vaknin et al. (1988) was applied to obtain CSD estimates at the superficial and bottom electrode contact points. The inverse CSD (iCSD) methods (Pettersen et al., 2006) are instead based on numerical inversion of the electrostatic forward model (analogous to Eq. 9), allowing for explicit incorporation of various assumptions on, for example, the underlying geometry of CSD sources. Here, two iCSD methods are considered: the δ-iCSD method assuming infinitesimally thin, circular disk-like current sources, and the spline-iCSD method, which assumes a cylindrical CSD distribution, yet spatially smooth in the depth direction (Pettersen et al., 2006). The assumed radius of the extent of current sources for the different iCSD methods is throughout this paper denoted *r*_CSD_.

For spatial filtering of the calculated CSDs (ground-truth CSD as well as reconstructed CSD from LFPs) we used normalized, discrete zero phase-shift finite impulse response filters of order *N* with Gaussian kernels with filter coefficients given by the following (Eq. 10):

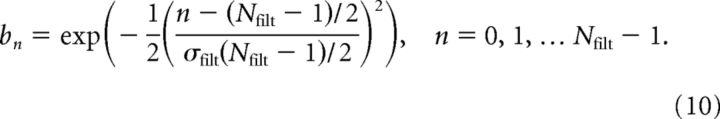
 The filter coefficients *b_n_* were normalized by the sum of the elements before filtering. For the standard-resolution CSD signals *N*_filt_ = 3 and σ_filt_ = 1 were used, here resulting in *b* = [*b*_1_, *b*_2_, *b*_3_] ≈ [0.274, 0.452, 0.274]. The only exception was the spline-iCSD estimates (and corresponding ground-truth CSDs) where the spatial sampling interval was only 20 μm, and *N*_filt_ = 19 and σ_filt_ = 5 were used. Note that CSD estimates were obtained from raw LFPs. Spatial filters were applied as a discrete convolution along the spatial axis. No temporal filters were applied.

For our CSD estimates, we have made implementations of each method in the Python programming language publicly available on Github (standalone package: https://github.com/espenhgn/iCSD; as part of the Elephant Electrophysiology Analysis Toolkit: https://github.com/NeuralEnsemble/elephant; RRID:SCR_003833).

##### Ground-truth CSD.

For testing of the accuracy of CSD estimation methods, we computed the ground-truth CSD from the transmembrane currents from all compartments of all neurons in the postsynaptic populations. With *M* model neurons (indexed by the integer variable *j*) in the population, each divided into *N* cylindrical compartments with lengths Δ*s_i_* and transmembrane currents *I_j_*_,_*_i_*, the ground-truth CSD in a volume element Ω*_k_* with volume *V*_Ω_*k*__ was calculated as follows (Eq. 11):

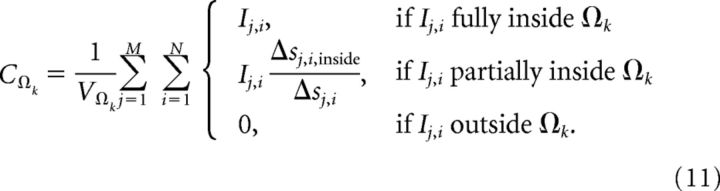
 Here Δ*s_j_*_,_*_i_*_,inside_ denotes the length of the line source (along the compartment cylinder axis), which is located inside the volume Ω*_k_*. The subscript index *k* used here denotes one of *K* cylindrical CSD volumes stacked along the vertical *z*-axis. In the present application, we calculated the ground-truth CSD averaged over vertically oriented cylindrical volumes so that (Eq. 12)

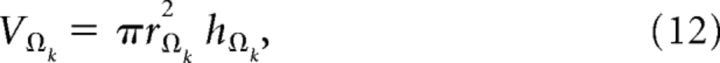
 where *r*_Ω_*k*__ is the cylinder radius, and *h*_Ω_*k*__ is the corresponding height. Two variants of the ground-truth CSD were considered, one with a spatial resolution of *h*_Ω_*k*__ = 100 μm, and another with *h*_Ω_*k*__ = 20 μm for validation of the spline-iCSD method. The values of *r*_Ω_*k*__ were set explicitly to the typical radius of the synaptic input projection, i.e., *r*_Ω_*k*__ = *r*_syn_ for synaptic projections with the spherical or cylindrical geometries.

As depth-resolved CSD estimates **C**_est_ = [*C*(*z*_1_, *t*), *C*(*z*_2_, *t*), …, *C*(*z_K_*, *t*)] with the different iCSD methods may depend on the source distribution radius *r*_CSD_, optimal values of *r*_CSD_ in the iCSD estimates were assessed by minimizing the least-square difference summed over all matrix elements (Eq. 13):

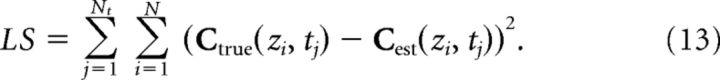
 We evaluated the expression across all *N_t_* time-steps and all *N* channels, with *r*_CSD_ in 5 μm increments up to 500 μm, to assess the optimal value. **C**_true_ = [*C*_Ω_1__(*t*), *C*_Ω_2__(*t*), …, *C*_Ω_*K*__(*t*)] was here the ground-truth CSD as assessed above.

Similarly we also computed Pearson product–moment correlation coefficients, denoted *cc*, between **C**_true_ and **C**_est_ to quantify similarity while ignoring overall signal amplitudes. We first flattened these matrices to vectors of length *N_t_* · *N*, here denoted *Ĉ*_true_ and *Ĉ*_est_, and used the following definition:

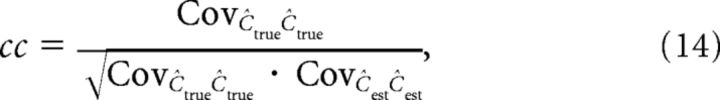
 where Cov denotes covariances.

##### Current-source density and potentials of concentric spherical shells.

In addition to calculating the ground-truth CSD in cylindrical volumes, we similarly (to Eq. 11) computed the spherically averaged CSD by summing transmembrane currents within *K* concentric spherical shells with thickness Δ*r* = 20 μm from the center of each population, divided by the corresponding volumes defined as follows (Eq. 15):

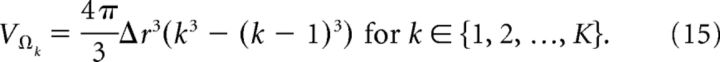
 Assuming homogeneity within each shell, the CSD as function of radius *r* is then a piecewise constant function *C*_CSD_(*r*) with no angular variation. An infinitesimally thin shell at radius *r* and thickness ∂*r* has then the net current ∂*i*_net_(*r*) = 4π*r*^2^*C*_CSD_(*r*)∂*r*. However, for illustration purposes, we plot the net current as a function of outer shell radius defined as Δ*i*_net_(*k*Δ*r*) = 4π*r*^2^*C*_CSD_(*k*Δ*r*)Δ*r* for *k* ∈ {0,1,…, *K*}. With spherical symmetry, the Poisson equation ∇^2^φ = −*C*_CSD_/σ (Nunez and Srinivasan, 2006) reduces to the following (Eq. 16):

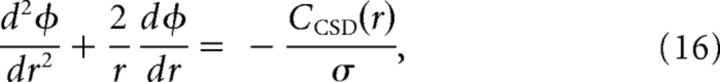
 where φ denotes the electric potential, and σ denotes the electrical conductivity. As the radial electric field is given by *E* = −*d*φ/*dr* in electrostatic theory, Eq. 16 can be rewritten as the following first-order ordinary differential equation (ODE; Eq. 17):

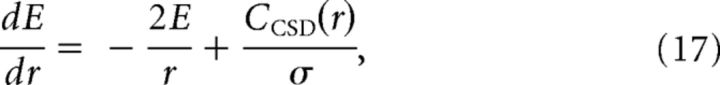
 which we solved numerically with the boundary condition *E*(0) = 0 and maximum radial step size of 1 μm up to a radius *r* = *K*Δ*r* = 500 μm, i.e., outside the outermost dendritic branch, using the function scipy.integrate.odeint. The electric potential is in general given as follows: φ(*r*) = const. − $\int_{0}^{r}E\left(r\prime \right)dr\prime$. Since both *C*_CSD_(*r*) (for lack of dendritic sources) and *i*_net_ (due to overall balance of current sinks and sources) are zero outside the region with dendrites, we conveniently defined φ(∞) = 0. We then computed the potential from the set of spherical shells with thickness Δ*r* as φ(*k*Δ*r*) = const. − ∑_*k*<*K*_ *E*(*k*Δ*r*)Δ*r* with the condition φ(*K*Δ*r*) = 0. Note that for our case of spherical symmetry, the condition φ(*K*Δ*r*) = 0 is analogous to the standard convention of assuming φ(*r* → ∞) = 0 when *K*Δ*r* is sufficiently large to encapsulate all transmembrane currents so that $\int_{0}^{K\Delta r}4\pi r^{2}C_{\text{CSD}}\left(r\right)dr=0$. In fact, in the present application of this scheme for computing electrical fields, this will be the case (with *r* = *K*Δ*r* = 500 μm).

##### Simulation platform.

The *in silico* modeling work presented here was fully incorporated in the Python programming language (http://www.python.org; RRID:SCR_008394; Langtangen, 2009). Simulations of extracellular potentials were facilitated by the package LFPy (http://LFPy.github.io; RRID:SRC_014805; Lindén et al., 2013), wherein the NEURON simulation environment was used to estimate transmembrane currents in each compartment (http://www.neuron.yale.edu; RRID:SCR_005393; Carnevale and Hines, 2006; Hines et al., 2009). Time-consuming simulation steps (e.g., calculations of extracellular potentials) were executed in parallel, facilitated by the multiprocessing module in Python. The implementations of the tCSD and iCSD estimation methods were ported from the Matlab toolbox CSDplotter (Pettersen et al., 2006) to Python. Least-square fitting of time constants and amplitudes to synapse currents, EPSPs, and EPSCs were treated as general constrained, nonlinear optimization problems, using the package OpenOpt.NLP (Kroshko, 2007). Remaining numerical analysis, filtering, and plotting relied on numpy, scipy, and matplotlib (http://www.scipy.org; RRID:SCR_008058; Jones et al., 2001; Hunter, 2007). Numerical solution of first-order ODEs were done using lsoda from the Fortran library odepack through scipy.integrate.odeint. All simulation and plotting code for this study is available for cloning on GitHub (http://www.github.com/LFPy/monosynaptic_LFP).

## Results

Figure 1 illustrates schematically the key model components set up to mimic the experimental set-up for probing the LFP signature of monosynaptic inputs to layer 4 in the sensory cortex (Swadlow et al., 2002). A laminar multielectrode records the electrical potentials vertically through the cortical lamina, including layer 4, while an electrode outside the thalamic cell (el.e) records spiking activity allowing the stLFP in the cortex to be computed (Fig. 1*A*).

Note that we do not explicitly model the TC neurons. Instead the arrival of the spike of the TC neuron onto the cortical cells is imposed as an abrupt activation of the synaptic conductances. To mimic the experimental situation, we have in the model imposed a general time delay τ_delay_ = 1.4 ms of the onset of the synaptic conductances compared with the “imagined” TC spike occurring at *t* = 0 ms.

Figure 1*A* also illustrates the intracellular patch-clamp electrodes (el.i) in layer-4 RS and FS cells used to measure EPSCs and EPSPs in previous experiments by Beierlein et al. (2003) and Hull et al. (2009). Results from these are here used to set the model parameters. An example of populations of multicompartmental RS and FS cells equipped with synapses, with a laminar multielectrode penetrating the center of the population (only electrode contacts are depicted), is shown in Fig. 1*B*. Morphological reconstructions of the RS and FS cells used to build the populations are rendered in Fig. 1*C*, with all possible postsynaptic target compartments marked in red.

In the experimental measurements, great care was taken to obtain reliable estimates of the stLFP signatures of individual incoming spikes from single TC neurons and avoiding contamination in the stLFP signature from postsynaptic spikes. This was achieved by averaging LFPs over thousands of spontaneous TC spikes to cancel out LFP contributions from ongoing cortical activity and avoid effects from correlated firing of neighboring TC neurons (Swadlow et al., 2002). The rapid-onset responses from different TC neurons measured with the same cortical electrode have been shown to be markedly different in terms of latency and depth profiles, and individual responses have been shown to vary little between different awake brain states (Stoelzel et al., 2009), which suggest that contamination of stLFPs by correlated activity is small. In the present modeling, effects of ongoing activity are absent per construction as there are no intracortical connections in the model and only a single spike from a single TC neuron drives the cortical cell population. Thus, model predictions of experimentally recorded stLFPs can be obtained by a single simulation run of the model. Further, spiking does not occur because the postsynaptic cortical model neurons do not have active conductances.

In the following three sections, we demonstrate that our model can indeed account for measured stLFPs both in the rabbit somatosensory cortex (Swadlow et al., 2002) and the rabbit visual cortex (Stoelzel et al., 2008). Subsequently we explore the effects of different synapse projection patterns on the stLFP (see below, stLFP signature depends on spatial extension of synaptic projection). The lateral “locality” of the stLFP (i.e., the spread of this synaptic LFP signature in the lateral directions) is addressed after that (see below, Strong stLFP signal only inside synaptic target regions and Biophysical origin of local stLFP signal) and we show that the stLFP signal is only strong when recorded inside the synaptic target region. In the subsequent section (see below, Only iCSD methods can give reliable CSD estimates), our model results are used as ground-truth to assess the validity of various methods for CSD estimation from the LFP in the present situation, and we find that only iCSD methods (Pettersen et al., 2006) can provide reliable CSD estimates from stLFP signatures. Finally, in the last section (see below, CSD analysis of experimental stLFPs), we use the insights obtained here to provide improved CSD estimates from the experimentally measured stLFP responses of monosynaptic connections.

### Contribution to stLFP signature from RS-cell population

**Figure 2.**
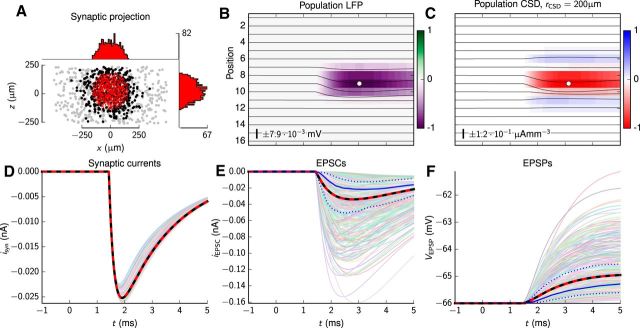
Schematic representation of the RS-cell population and their postsynaptic responses. The responses are obtained for 470 postsynaptic cells and 1311 dendritic synapses corresponding to the putative monosynaptic connection of a TC afferent onto RS cells. Synapses were allowed within a sphere with radius *r*_syn_ = 165 μm, vertically offset by μ*_z_* = 35 μm (compare Eq. 4). ***A***, Scatter plot of synaptic and somatic locations within the population. Gray dots denote somas of neurons without TC synapses; black dots denote somas of postsynaptic cells; red dots denote synapse locations. The histograms on top and to the right show the spatial distribution of synapse locations along the horizontal *x*-axis and vertical *z*-axis, respectively. The single tick label of each histogram denotes the maximum synapse count within 10 μm bins along the depicted axes. ***B***, LFP responses from the 16-channel laminar electrode shown as image and line plots. The white circle marker denotes the position and time of the global LFP minimum relative to the image plot (electrode position 9 and *t* = 3.03 ms). ***C***, Ground-truth CSD for the postsynaptic response. The global CSD minimum is at electrode position 9 and *t* = 3.13 ms. The default spatial filtering procedure was applied. ***D***, Synaptic input currents: traces of individual synaptic currents (thin colored lines), averaged current (black line), and least-square fit of the two-exponential function to the averaged current (dashed red line). ***E***, Somatic EPSCs recorded in the somas of postsynaptic cells with the somatic voltages clamped to the resting potential of each neuron: individual EPSC traces (thin colored lines), averaged EPSC (black line), and least-square fit of the two-exponential function to averaged EPSC (dashed red line). The solid blue line denotes the median EPSC, and the dotted blue lines denote the lower and upper quartile (25 and 75% percentile) of the data. ***F***, Somatic EPSPs recorded in the somas of postsynaptic cells: individual EPSP traces (thin colored lines), averaged EPSP (black line), and least-square fit of the two-exponential function to averaged EPSP (dashed red line). The solid blue line denotes the median EPSP, and the dotted blue lines denote the lower and upper quartile (25 and 75% percentile) of the data. Note that in ***D–F***, the black lines almost completely overlap the red lines. In ***B*** and ***C***, the number and scale bar describe the amplitude of the line plots. The number also gives the LFP/CSD values corresponding to ±1 of the color bar.

Figure 2 shows the postsynaptic responses due to monosynaptic input from a TC cell onto the population of RS cells, including LFPs, (ground-truth) CSDs, and intracellular responses (EPSPs, EPSCs). A spherical spatial distribution of synapses with radius *r*_syn_ = 165 μm was positioned with a positive vertical offset (μ*_z_* = 35 μm) on to a cylindrical population of RS cells, resulting in the distribution of synaptic locations depicted in Figure 2*A*. While the synapses (red dots) themselves were confined to dendritic compartments within a sphere, some of the somas of the neurons receiving these synapses (black dots) happened to be outside the spherical target region. The histograms in Figure 2*A* demonstrate that the synapse placement procedure resulted in an approximately homogeneous density of synapse sites within the RS population.

An initial observation is that the set of synaptic currents recorded at the different synapse sites deviate little from the mean synaptic current (Fig. 2*D*), implying that responses at the synapse sites remained almost linear, i.e., changes in membrane voltages around the synaptic inputs were too small to appreciably change the synaptic driving force (i.e., the potential difference *V*_m_ − *E*_syn_) at the conductance-based synapses. In contrast, the EPSCs (with soma potentials clamped at membrane reversal potential) and EPSPs recorded in the soma of each postsynaptic cell display large variabilities both in terms of their amplitudes and temporal shapes (Fig. 2*E*,*F*). The inherent variability in somatic EPSPs and EPSCs is illustrated in these panels by the median (solid blue line) and upper and lower quartiles (75 and 25% percentiles, dotted blue lines) computed from all single-cell observations. As we assumed the same maximum synaptic conductance *g*_max_ for all synapses, the observed variability reflected the different locations of the individual synapses. Such variability occurs due to different low-pass filtering of synaptic input currents along the dendritic cables between the synapse sites and somas, and summation of individual responses whenever several synapses were present on individual neurons. The mean EPSC and EPSP (Fig. 2*E*,*F*, thick black lines) were well fitted by a double-exponential function (dashed red lines). Further, the fitted values of the function parameters listed in Table 3 were found to be in good qualitative agreement with the parameters found from fitting the same function to the experimental EPSPs by Beierlein et al. (2003) and EPSCs by Hull et al. (2009; Table 2).

With model predictions for intracellular recordings confirmed to be in accordance with experiments, we next turned to the computed LFP signatures stemming from the monosynaptic thalamic input. The resulting LFP is found to be largely confined to the region around the synaptic inputs, i.e., only giving sizable signals at electrode contacts 7–10 inside or near the edge of the synaptic input region (Fig. 2*B*). Further, the chosen value of *r*_syn_ = 165 μm gives a vertical spread of LFP signatures comparable to results reported by Swadlow et al. (2002) and Stoelzel et al. (2008). Incidentally, this radius is comparable to the vertical and horizontal spread of thalamic afferents in the cat visual cortex (Freund et al., 1985) and in the rat somatosensory cortex (Oberlaender et al., 2012). These two anatomical studies also demonstrated that different thalamic afferents targeting the same cortical region display great variability in terms of their innervation pattern, e.g., some appear to preferentially target different regions within layer 4 (and also layer 6) with varying levels of branching. For example Figure 5 of Oberlaender et al. (2012) suggests that the highest density of connections made by individual ventral posteromedial (VPM) neurons occur within a volume ∼200–400 μm in width and height, in line with our chosen *r*_syn_, although significant axon-to-axon variability was observed. To agree better with the experimental stLFP results from Swadlow et al. (2002), a small vertical offset of μ*_z_* = 35 μm was chosen. This shifted the (hypothetical) maximum of the LFP response to be slightly above electrode 9 so that the maximum LFP signal of electrode 8 became larger than the corresponding LFP signal at electrode 10. The LFP minimum at electrode 9 for the example in Figure 2*B* was −7.9 · 10^−3^ mV occurring at a latency of *t* = 3.03 ms.

The results in Figure 2*B* were obtained assuming an electrode contact radius of *r*_contact_ = 15 μm (Stoelzel et al., 2008). We also tested other contact sizes (i.e., *r*_contact_ = 7.5, 11.5, and 20 μm), but essentially got the same results both in terms of LFP magnitude and temporal frequency content (data not shown).

In the experimental situation, each TC neuron will have a unique projection pattern, that is, a unique set of postsynaptic neurons where each postsynaptic neuron is targeted by a unique set of synapses (from the single presynaptic neuron) on particular positions of the dendrites. This will give each TC neuron a unique stLFP signature, and there will thus be variability in the recorded stLFP signatures (Swadlow et al., 2002). In the model, we only include two distinct model neurons, one RS neuron and one FS neuron, in the receiving populations. Likewise, the shapes and sizes of the synaptic conductances are fixed. However, both the detailed positions and rotation angle of each postsynaptic neuron are chosen randomly for each simulation. Further, the detailed number and positions of the synapse are also chosen randomly according to prescribed statistical rules. As in experiments where there are variations in the stLFP signatures of different TC neurons, there will thus also be variations in the resulting stLFP signatures between each model instance.

The results in Figure 2 correspond to a single model instance. To quantify the variability between model instances, we tested the effect of statistical variations in cell locations, alignments, and synapse positions by redoing the simulations with different seeds to the pseudorandom number generator. For a sample of 10 different model instances, we found an LFP minimum value of (−7.7 ± 0.7) · 10^−3^ mV occurring at a latency of *t* = 3.05 ± 0.08 ms at electrode position 8.9 ± 0.3 (data not shown).

The depth-resolved ground-truth CSD is as expected more localized in the vertical directions than the LFP (Fig. 2*C*). Here, the resulting current sink stemming from the synaptic input currents themselves is mostly confined within a vertical depth of 200–300 μm. The current sources above and below the main synaptic input sites, stemming from return currents associated with these synaptic inputs, are seen to have much smaller magnitudes than the sinks. This indicates that these return currents have a larger radial spread than the synaptic sink currents due to dendrites and somas located outside the synaptic target region (compare Fig. 1*B*). Thus, while the CSD summed across the entire 3D volume by necessity must add up to zero (Pettersen et al., 2006, 2012), the CSD summed along the axis of a spatially restricted cylinder does not necessarily sum to zero. Like our findings for the LFP, we found little variability in the CSD minima when changing the random seed in our simulations across 10 model instances: the CSD minimum value was found to be −0.12 ± 0.01 μAmm^−3^ occurring at a latency of *t* = 3.10 ± 0.03 ms at electrode position 9.0 ± 0.0 (data not shown).

The temporal envelopes of the LFPs and CSDs are seen in Figure 2*B*,*C* to be similar to the temporal profiles of the mean EPSC, i.e., much faster than the temporal profile of the mean EPSP. This demonstrates that the LFP is more closely related to the synaptically driven transmembrane currents than to the membrane potential (Lindén et al., 2010; Pettersen et al., 2012).

### Contribution to stLFP signature from FS-cell population

**Figure 3.**
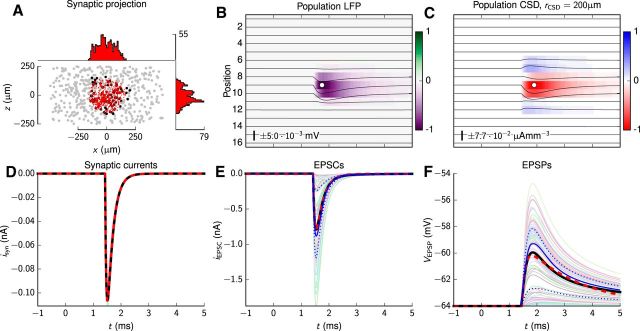
Schematic of the postsynaptic FS-cell population and their postsynaptic responses. These results were obtained from 88 postsynaptic cells and 932 somatic and proximal synapses. Synapses were located in a sphere with radius of *r*_syn_ = 165 μm, but with no vertical offset (μ*_z_* = 0 μm). ***A***, Scatter plot of synaptic and somatic locations. Gray dots denote somas of neurons without synapses; black dots denote neurons with synapses; red dots denote synaptic locations. The histograms on top and to the right show the spatial distribution of synapse locations along the horizontal *x*-axis and vertical *z*-axis, respectively. The single tick label of each histogram denotes the maximum synapse count within 10 μm bins along the depicted axes. ***B***, LFP responses from the 16-channel laminar electrode shown as image and line plots. The white circle marker denotes the position and time of the global LFP minimum relative to the image plot (electrode position 9 and *t* = 1.75 ms). ***C***, Ground-truth CSD for the postsynaptic response. The maximum and minimum values in the color plot are ±0.077 μA/mm^3^. The global CSD minimum is at electrode position 9 and *t* = 1.90 ms. The default spatial filtering procedure was applied. ***D***, Synaptic input currents: traces of individual synaptic currents (thin colored lines), averaged current (black line), and least-square fit of the two-exponential function to the averaged current (dashed red line). ***E***, Somatic EPSCs recorded in the somas of postsynaptic cells with the somatic voltages clamped to the resting potential of each neuron: individual EPSC traces (thin colored lines), averaged EPSC (black line), and least-square fit of the two-exponential function to the average EPSC (dashed red line). The solid blue line denotes the median EPSC, and the dotted blue lines denote the upper and lower quartile of the data. ***F***, Somatic EPSPs recorded in the somas of postsynaptic cells: individual EPSP traces (thin colored lines), averaged EPSP (black line), and least-square fit of the two-exponential function to the average EPSP (dashed red line). The solid blue line denotes the median EPSP; the dotted blue lines denote the upper and lower quartile of the data. In ***B*** and ***C***, the number and scale bar describe the amplitude of the line plots. The number also gives the LFP/CSD values corresponding to ±1 of the color bar.

We next repeated the simulation experiment for a population of FS cells, with one-quarter of the cell density compared with RS-cell population (1000 FS cells vs 4000 RS cells), in accordance with earlier literature estimates that ∼15–25% of cortical neurons are GABAergic (Swadlow, 2003). As for the RS-cell population, synapses are placed within a sphere with radius *r*_syn_ = 165 μm. However, unlike for the RS population, for the FS population the synapse distribution is centered in the population (μ*_z_* = 0 μm), and synapses are placed only on the somatic and proximal dendrite compartments. Due to the high probability of connection onto somatic compartments, most targeted neurons have their somas contained in the synaptic target volume (Fig. 3*A*).

The individual synaptic currents in the FS cells exhibit even smaller deviations from the mean synaptic current than in the RS cells (Fig. 3*D*), while the temporal profiles of EPSCs and EPSPs recorded in the somas show more variation between the neurons (Fig. 3*E*,*F*). All synapses are positioned on or close to the soma, resulting in little variation in the dendritic filtering, so the variation in the EPSC and EPSP amplitudes are due to the different number of synapses placed on the various FS neurons. As for the RS cells, the mean EPSC and EPSP are well fitted by the two-exponential function in Equation 2 (Fig. 3*E*,*F*, compare thick black, dashed red lines; *R*^2^ ≈ 1). For the FS cells, the fitted values of the function parameters, listed in Table 3, are seen to be in good qualitative agreement with the parameters found from fitting the same function to the experimental data by Beierlein et al. (2003) and Hull et al. (2009), summarized in Table 2.

Although the synaptic inputs are preferentially onto somas and proximal dendrites and thus more focal than the synaptic input onto the RS cells, the resulting vertical spreads of LFPs and CSDs (Fig. 3*B*,*C*) are similar to those contributed by the RS cells (Fig. 2*B*,*C*): the LFP negativity associated with the FS cells is found across ∼5 channels (i.e., ∼500 μm); the CSD negativity associated with the FS cells is found across ∼3 channels. Further, the peak LFP and CSD amplitudes from the projections onto the FS-cell population are also of the same order of magnitude, i.e., 60–70% of those for the RS-cell population. For 10 different model instances using different random seeds, we found the minimum value of LFPs to be (−4.6 ± 1.2) · 10^−3^ mV occurring at a latency of *t* = 1.81 ± 0.06 ms on electrode position 9.1 ± 0.5. Similarly, the CSD minimum value was found to be (−6.9 ± 1.3) · 10^−2^ μAmm^−3^ occurring at a latency of *t* = 1.89 ± 0.05 ms on electrode position 9.0 ± 0.0 (data not shown).

Note that larger cross-trial variabilities in maximum LFP magnitude were observed for the FS-cell population than for the RS-cell population both in absolute and relative terms, i.e., (4.6 ± 1.2) · 10^−3^ mV for the FS population versus (7.7 ± 0.7) · 10^−3^ mV for the RS population. The same was observed for the variability in maximum CSD magnitude, i.e., (6.9 ± 1.3) · 10^−2^ μAmm^−3^ versus 0.12 ± 0.01 μAmm^−3^. This is as expected given that there is a factor four fewer FS neurons than RS neurons in the postsynaptic population and thus larger statistical variation (this explanation was supported by separate simulations with 500 rather than 1000 FS neurons, which as predicted gave an even larger cross-trial variability; data not shown).

A striking feature is the much shorter latency to peak for the FS population (*t* = 1.81 ± 0.06 ms) than for the RS population (*t* = 3.05 ± 0.08 ms). Note that in both cases, 1.4 ms of this latency of the stLFP signal stems from the imposed delay of 1.4 ms in synaptic activation following a spike in the TC neuron. The shorter latency for FS neurons than for RS neurons stems from the shorter membrane time constants assumed for FS neurons compared with RS neurons (i.e., 5 vs 10 ms; compare Table 1). Moreover, synaptic input currents are also faster for FS neurons than for RS neurons (compare Figs. 3*D*, 2*D*). The faster time course is reflected not only in a shorter latency of the stLFP (and corresponding stCSD) profiles for the FS cells compared with the RS cells, but also in shorter overall duration, i.e., temporally sharper envelopes of the LFP and CSD signal (Fig. 3*B*,*C*).

### Combined stLFP signature from RS and FS cells resembles experiments

As seen above, the FS-cell population also appears to give a substantial contribution to the stLFP signature, with peak LFP and CSD magnitudes more than half of those contributed by the RS-cell population. This may at first glance be surprising as there are four times as many RS cells than FS cells in our model. However, other factors contribute as well, in particular the larger average number of converging synaptic inputs onto the FS cells in the postsynaptic population compared with onto the RS-cell population (by setting *n̄*_syn_ = 15 vs *n̄*_syn_ = 7 for the FS-cell and RS-cell population, respectively; see Materials and Methods), but also differences in maximal synaptic conductances, time constants, and synapse locations.

While peak amplitudes of the contribution to the LFP were comparable, the LFP response from the FS-cell population had a sharper temporal profile than from the RS-cell population. These differences in responses stemmed from the shorter duration of synaptic input currents, briefer membrane time constants of FS cells compared with RS cells, and the more soma-proximal convergence of synaptic inputs from TC neurons onto FS cells than RS cells (see Materials and Methods, Placement of thalamocortical synapses onto populations). This finding may explain a feature seen in several experimental recordings of the stLFP signature of monosynaptic thalamic input onto sensory cortices: the short-latency postsynaptic part of the signature (stemming from monosynaptic effects) appears to consist of two separate components, sometimes positioned at different cortical depths (Swadlow et al., 2002; Stoelzel et al., 2008). This hints that separate postsynaptic populations recruited by each thalamic afferent may contribute to the observed stLFP signatures.

**Figure 4.**
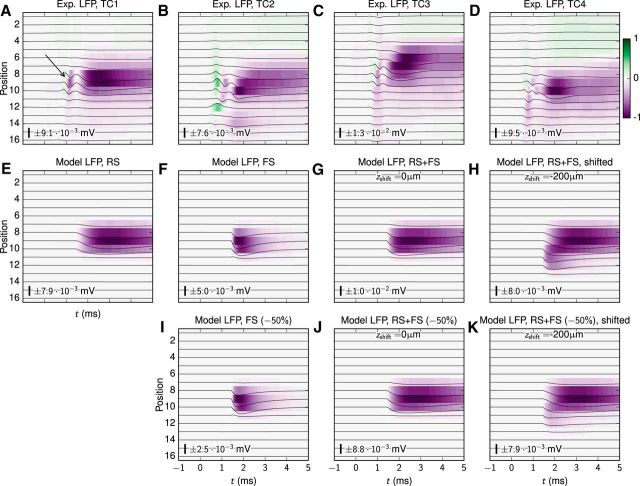
Model versus experimental stLFPs. Comparison of stLFP recordings obtained *in vivo* with model LFPs from the RS-cell and FS-cell populations, independently and with superposition of the contributions from each model population. ***A***, Postsynaptic LFP responses from an individual TC afferent in the rabbit somatosensory (S1) cortex, from Swadlow et al. (2002, their Fig. 1*B–N1*). The arrow points to the short presynaptic contribution to the LFP. ***B***, Postsynaptic LFP responses of another TC afferent projecting to S1 from Swadlow et al. (2002, their Fig. 1*B–N2*). ***C***, stLFP responses from a LGN afferent obtained in rabbit visual cortex (V1), from Stoelzel et al. (2008, their Fig. 1*E*). ***D***, Postsynaptic responses of another LGN afferent recorded in V1 from the same study (Stoelzel et al., 2008, their Fig. 2*A1*). ***E***, Postsynaptic LFPs from the model RS-cell population (see Results, Contribution to stLFP signature from RS cell population). ***F***, Postsynaptic LFPs from the model FS-cell population (compare Results, Contribution to stLFP signature from FS cell population). ***G***, Linear superposition of LFP contributions from the model RS-cell and FS-cell populations. ***H***, Linear superposition of LFP contributions from the model RS-cell and FS-cell populations, but with the FS-cell contribution spatially offset vertically by −200 μm. ***I–K***, Same as ***F–H***, but with the FS-cell population contribution to the LFP reduced by 50%. In all panels, the number and scale bar describe the amplitude of the line plots. The number also gives the LFP values corresponding to ±1 of the color bar.

Here, we investigate to what extent these experimentally recorded stLFP signatures can be accounted for by a model where single thalamic cells impinge on the RS-cell and FS-cell populations, i.e., whether these signatures can be mimicked in our model by a sum of the two individual population contributions investigated above. In Figure 4*A–D*, we show four monosynaptic stLFP responses previously reported by Swadlow et al. (2002) and Stoelzel et al. (2008). The first two responses (Fig. 4*A*,*B*) were assessed by recording spontaneous firing activity in two different TC neurons (TC1, TC2) targeting the topographically aligned somatosensory cortex (S1) in awake rabbits (Swadlow et al., 2002), while the latter two responses (Fig. 4*C*,*D*) were recorded similarly from the rabbit visual cortex (V1; TC3, TC4) with thalamic afferents from the LGN (Stoelzel et al., 2008). Even though these postsynaptic LFP responses were recorded in different cortical areas, the results have several similar characteristics: (1) postsynaptic signals are preceded by fast transients caused by presynaptic action-potential propagation in the thalamic afferent with a distinct (temporal) narrow gap between the presynaptic and postsynaptic LFP responses (Stoelzel et al., 2008; Jin et al., 2011; Fig. 4*A*, arrow; note that we do not model this presynaptic LFP signature here); (2) three of the responses (TC1, TC3, TC4) have very similar spatial LFP profiles (the TC2 afferent was likely presynaptic to both layer-4 cells and layer-6 cells with overlapping postsynaptic LFP signatures; Swadlow et al., 2002); and (3) even though peak LFP amplitudes may be located at different cortical depths, the postsynaptic LFP signals reach maximum magnitude quickly (*x* ≲ 200 ms) followed by a decay of longer duration.

The second row of panels in Figure 4 illustrates how the characteristic features of the recorded stLFP signatures (particularly focusing on TC1 and TC2) can be accounted for by summation of the contributions from our model RS-cell and FS-cell stLFP signatures shown in Figures 2 and 3, respectively. As shown in Figure 4*E*, the LFP contribution from the RS-cell population already corresponds quite well with the TC1 response in Figure 4*A* in terms of vertical spatial spread and LFP magnitude. However, close inspection reveals that the temporal response of the model LFP is a bit longer-lasting, but this disagreement could be remedied by adding the model contribution from the FS-cell population in Figure 4*F*, giving the net summed LFP signature depicted in Figure 4*G*. To make a quantitative comparison between experimental data and model results, we concatenated the signals on all electrode contacts and computed the pairwise correlation coefficients [Pearson product–moment correlation coefficients (*cc*)] between the resulting vectors. The correlation between the model results for the RS population and the TC1 stLFP (Fig. 4*A*; *cc* = 0.940) was higher than for TC2 (Fig. 4*B*; *cc* = 0.846). The stLFP of the FS-cell population was much less correlated with the TC1 and TC2 stLFPs (*cc* = 0.653 and *cc* = 0.706). However, with superimposed RS-cell and FS-cell stLFPs (Fig. 4*G*), the deviations between the model and the experimental TC2 stLFP were substantially reduced (*cc* = 0.878). The correlation between the superimposed RS-cell and FS-cell LFP and the TC1 neuron remained similar (*cc* = 0.938). Note that these correlation coefficients were computed using experimental data that also included the presynaptic LFP components of TC1 and TC2 stLFPs. These presynaptic LFP components were not included in the model because the transmembrane currents in the axonal boutons were not included. Thus, the overall agreement with the model (i.e., the value of the correlation coefficient) was somewhat reduced.

The experimental signatures of TC2 in Figure 4*B* (and also for TC3 in Fig. 4*C*) have an upward-moving LFP negativity, i.e., the onset of the postsynaptic LFP response is slightly delayed at the more superficial electrode contacts. This feature can be naturally emulated in our model by assuming that TC afferents preferentially target excitatory and inhibitory cells at different cortical depths. As an example, Figure 4*H* shows the net summed LFP signature when the FS-cell contribution to the LFP was vertically shifted 200 μm downwards, revealing this feature. As expected, spatially shifting the FS contribution upwards increased the similarity with the TC2 stLFP (*cc* = 0.891), but simultaneously reduced the agreement with the TC1 stLFP (*cc* = 0.922).

Recent studies from the rat barrel cortex have pointed to a smaller fraction of inhibitory neurons in layer 4 than the 20% assumed in Figure 4; more on the order of 10% (Meyer et al., 2011). The specific experiments targeted in our present modeling are from rabbits rather than rats, and it is unclear how the ratio between inhibitory and excitatory neurons generalizes between species. Nevertheless, in Figure 4*I–K* we illustrate the dependence of the stLFP signature on the assumed fraction of inhibitory neurons by showing results analogous to those in Figure 4*F–H* for the case where the FS contribution to the overall signature is reduced by half, mimicking a situation with an inhibitory-neuron fraction of ∼10%. As observed, the predicted total stLFP signatures for the lower fraction (Fig. 4*J*,*K*) are quite similar in appearance to the corresponding results for the original fraction (Fig. 4*F–H*). While the amplitudes are slightly reduced, our overall qualitative conclusions are unchanged.

Note that LFP signatures in the deeper layers, as seen for the experimental TC2 cell in Figure 4*B*, are not present in our model results, reflecting that neither layer-5 nor layer-6 cells were included in our model. TC afferents may also synapse onto these infragranular cell types within the boundaries of layer 4 (Binzegger et al., 2004; Oberlaender et al., 2012) and contribute to the LFP response in layer 4 and above. However, here we assumed that these contributions were small compared with the contributions from the layer-4 cells.

### stLFP signature depends on spatial extension of synaptic projection

**Figure 5.**
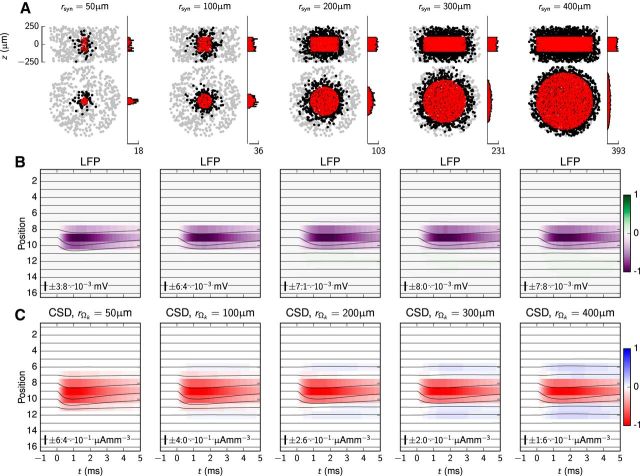
Effect of lateral extent of cylindrical synaptic target region on LFPs and CSDs in populations of RS cells. Synapse parameters were set to their default values, except that synapse activation occurred at *t* = 0 ms. The height and corresponding radius of the regions of synaptic inputs was *h*_syn_ = 200 μm and *r*_syn_ ∈ {50, 100, 200, 300, 400} μm. No vertical offsets were applied. Respective postsynaptic soma counts: {68, 209, 666, 1301, 2088}. Synapse counts: {115, 438, 1758, 3972, 6886}. ***A***, Scatter plots showing the synaptic distributions projected in the *xz* and *xy* planes, respectively, with corresponding histograms of synaptic locations. Gray dots denote somas of neurons without synapses; black dots denote postsynaptic neurons; red dots denote the synapse locations. The histograms show the spatial distribution of synapse locations along the *x*-axis and *z*-axis, respectively. The single tick label denotes the maximum synapse count within 10 μm bins along the depicted axes. ***B***, Effect on synaptic distribution radius on postsynaptic LFP sinks. ***C***, Effect of synaptic distribution radius on ground-truth CSD, the domain radius *r*_Ω_*k*__ is now the same as the synaptic distribution radius, *r*_syn_. In ***B*** and ***C*** the number and scale bar describe the amplitude of the line plots. The number also gives the LFP/CSD values corresponding to ±1 of the color bar.

The above results were based on a biologically plausible, yet uncertain assumption about the size and geometry of the synaptic target region (but see Freund et al., 1985; Oberlaender et al., 2012). To investigate the effect of the lateral size of the synaptic region, we ran a series of simulations with cylindrical synaptic target regions onto the RS-cell population as described above. The cylindrical synaptic target region was concentric with the RS-cell population and had a fixed cylinder height of *h*_syn_ = 200 μm and radius *r*_syn_ that varied incrementally between 50 and 400 μm. No vertical offset was applied (μ*_z_* = 0 μm). The results for the LFP and ground-truth CSD, as well as plots depicting the spatial distributions of synapses, are shown in Figure 5.

The shape of the LFP signature as recorded in the center of the synaptic target region is observed to not vary much between the smallest (*r*_syn_ = 50 μm) and largest (*r*_syn_ = 400 μm) lateral target sizes considered (Fig. 5*B*). The maximum LFP magnitude is as expected smallest (−3.8 · 10^−3^ mV) for the smallest target radii, since small target regions make for fewer inputs in our model. However, for *r*_syn_ > 100 μm, the amplitudes do not vary much (between −7.9 · 10^−3^ and −6.5 · 10^−3^ mV). The observed minor reduction in LFP and CSD amplitude obtained with *r*_syn_ = 400 μm is here an artifact of the model setup: with our finite RS-population radius, the synaptic return currents were less likely to exit in the outward lateral directions, thus leading to more cancellations of CSDs and LFPs at the center of the cell population.

The computed ground-truth CSDs in Figure 5*C* show a more distinct variation with synaptic target size. While the CSD profile is dominated by the current sink (negative CSD) due to the synaptic currents themselves for *r*_syn_ = 50 μm, the CSD depth profiles for larger target regions exhibit substantial current source regions above and below the target cylinder. This trend toward more balance between sinks and sources with growing target regions is as expected, since in the (hypothetical) limit of an infinitely wide target region, i.e., r_syn_ → ∞, the summed sinks and sources will have to cancel exactly (Pettersen et al., 2006, 2008). We also note that the maximum CSD amplitude is substantially larger for the smallest synaptic target sizes *r*_syn_ than for the largest target sizes due to much smaller sink–source cancellations.

### Strong stLFP signal only inside synaptic target regions

So far we have only considered the situation where the laminar multielectrode penetrates the center of the synaptic target region, but in practical experimental situations this idealized situation is difficult to realize. We therefore next investigated how the measured LFP signature varied with the electrode offset, i.e., the lateral distance of the vertically oriented laminar electrode to the center of the synaptic target region. We further explored the effects on measured LFP signatures from the geometrical shape of the synaptic projection patterns by considering spherical, cylindrical, and Gaussian projection patterns.

**Figure 6.**
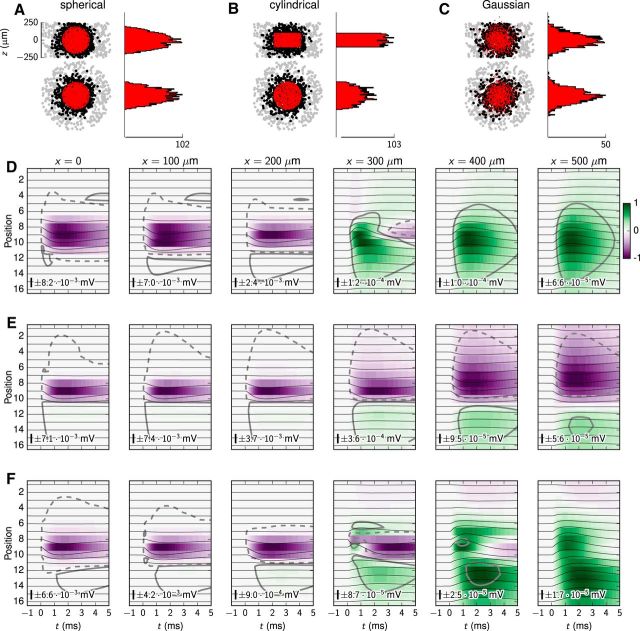
Effect of lateral electrode displacement on recorded LFPs in population of RS cells. Spatiotemporal postsynaptic LFP responses with lateral offsets of electrode device obtained for synaptic projection patterns with spherical, cylindrical, and Gaussian kernels, respectively, onto populations of RS cells. Synapse properties were similar to those of the default RS-cell population, but synapses were activated at *t* = 0 ms. ***A***, Postsynaptic population with a spherical synapse distribution (*r*_syn_ = 200 μm, μ*_z_* = 0 μm) consisting of 764 postsynaptic cells and 2355 synapses. The scatter plots show the synaptic distributions projected in the *xz* and *xy* planes, respectively, with corresponding histograms of synaptic locations. Gray dots denote somas of neurons without synapses; black dots denote postsynaptic neurons; red dots denote the synapse locations. The histograms show the spatial distribution of synapse locations along the *x*-axis and *z*-axis, respectively. The single tick label denotes the maximum synapse count within 10 μm bins along the depicted axes. ***B***, Population with a cylindrical synapse distribution (*r*_syn_ = *h*_syn_ = 200 μm, μ*_z_* = 0 μm) resulting in 666 postsynaptic cells with 1758 synapses. ***C***, Population with a Gaussian-type synapse distribution (σ*_x_* = σ*_y_* = σ*_z_* = 100 μm, μ*_z_* = 0 μm) consisting of 581 cells, with 1067 synapses. ***D***, Spatiotemporal LFP signatures for spherical synaptic distribution, at different lateral offsets along the *x*-axis. ***E***, Same as ***D***, but with the cylindrical synapse distribution. ***F***, Same as ***D*** and ***E***, but with the Gaussian synapse distribution. The number and scale bar inside ***D–F*** describe the amplitude of the line plots. The number also gives the LFP values corresponding to ±1 of the color bar. The gray isopotential contour lines denote LFP values of −2 · 10^−5^ mV (dashed) and 2 · 10^−5^ mV, respectively.

Figure 6 summarizes results obtained for the RS-cell population with both centered (*x* = 0) and five noncentered (*x* ∈ {100, 200, 300, 400, 500} μm) electrode positions. The three synaptic projection patterns were parameterized to have comparable spatial extensions, i.e., *r*_syn_ = 200 μm for the spherical function (Fig. 6*A*), *r*_syn_ = *h*_syn_ = 200 μm for the cylindrical function (Fig. 6*B*), and σ*_x_* = σ*_y_* = σ*_z_* = 100 μm for the Gaussian function (Fig. 6*C*). Each synapse projection pattern was centered in the origin, implying zero vertical offset (μ*_z_* = 0 μm).

An initial observation was that for the centered case and the two closest noncentered cases (*x* ∈ {100, 200} μm), there was little variation in the signature of the monosynaptic LFP response, both across the three synaptic distributions and across the electrode position (Fig. 6*D–F*, columns 1–3). In all situations, the LFP signature had characteristic negative potential values following synapse activation here set to be at *t* = 0 (i.e., no TC delay included). The isopotential lines in Figure 6*D–F* show that weakly positive LFPs may occur on top and below the main sinks (gray lines denoting potentials of 2 · 10^−5^ mV). Since the spherical synaptic distribution had a slightly larger effective “height” than the other synaptic distributions, the vertical spread of the signature was slightly greater (spanning contacts 7–11) for this case than for the other cases. Further, the peak LFP amplitudes also varied between the three synaptic distributions as the volume of each postsynaptic region, and thus the number of postsynaptic cells, was not identical. However, these small amplitude variations were inconsequential here, as we were mainly interested in the transition between recordings made inside and outside the synaptic target regions.

For distal noncentered electrode positions (*x* ∈ {300, 400, 500} μm), where the electrode was outside the synaptic target region, the situation changed. Here the LFP signature could clearly have both negative and positive signs, and the shapes also differed between the different synaptic projection patterns. More importantly, the LFP amplitudes were also very small, >1 order of magnitude smaller than for electrodes penetrating the population center (going from magnitudes of ∼10^−3^ mV for *x* = 200 μm at the edge of the target region to ∼10^−4^ mV and smaller for offsets of *x* ≥ 300 μm). Note that positive-valued LFPs occurred also for centered electrodes, here illustrated by the isopotential lines drawn in Figure 6*D–F*. The solid and dashed gray lines denote LFP values of +2 · 10^−5^ and −2 · 10^−5^ mV, respectively.

**Figure 7.**
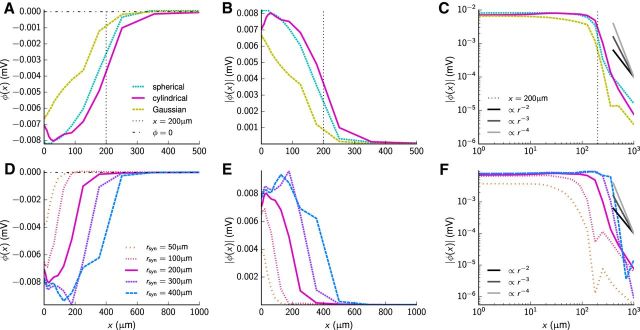
Dependence of LFP on lateral electrode position. ***A***, LFP as a function of lateral offset *x* for the three considered synapse projection patterns (spherical, cylindrical, Gaussian). In this and subsequent panels values are calculated at the time step corresponding to *t* = 1.5 ms. Further, the vertical dashed line at *x* = 200 μm denotes *r*_syn_ used for the cylindrical and spherical synapse distributions, and the horizontal dot-dashed line depicts the value 0. ***B***, LFP magnitude (absolute value) for results in ***A***. ***C***, Same as ***B*** with logarithmic axes. Power–law function decays (∝ *r*^−2^, *r*^−3^, *r*^−4^) depicted for comparison. ***D–F***, Same as ***A–C*** for cylindrical synapse projection patterns with varying radius *r*_syn_ as in Figure 5.

These findings imply that the LFP can be expected to have a dominant-negative deflection when the electrode is laterally positioned to be within the region of the synaptic input, while a much weaker LFP signal, positive or negative, is predicted when electrodes are positioned outside. To investigate this in more detail, we highlight in Figure 7 the sharp decay of the LFP signal that occurred outside the synaptic target region. In Figure 7*A*, the magnitude of the LFPs from the RS-cell populations at *t* = 1.5 ms is shown as a function of lateral distance of electrode position from the center of the synaptic target region, i.e., along the horizontal *x*-axis, for the spherical, cylindrical, and Gaussian synaptic patterns with the parameter *r*_syn_ = 200 μm. Shown both with linear (Fig. 7*B*) and logarithmic axes (Fig. 7*C*), the magnitude of the LFP is for all synaptic distributions seen to decay moderately inside the synaptic target region (i.e., for *x* ≲ 200 μm), but drops off sharply outside this region. Around the target region, the signal is seen to decay sharper than *r*^−4^ both for the spherical and cylindrical target patterns, while the decay for the Gaussian target pattern is slightly more rounded. Figure 7*D–F*, which shows results for the lateral dependence of the LFP for the cylindrical target-pattern case when the target radius *r*_syn_ is varied, demonstrates that this sharp decay also holds for the other radii considered (between 50 and 400 μm). We note, however, that the signal decay generally is sharpest for the largest synaptic target regions.

Our finding of a very attenuated stLFP signature outside the synaptic target region is in accordance with results from Swadlow and Gusev (2000), who measured stLFPs in neighboring barrel columns in the rabbit barrel cortex. There it was found that the stLFP signature was indeed very small when the LFPs were recorded in cortical columns, which were nonhomologous to the TC neurons used in the trigger averaging. In this situation, the TC cell in question primarily projects to a column next to where the LFP is recorded (i.e., the cortical recording electrode is placed outside the synaptic target region), so that a very small stLFP signal can be expected based on our modeling results. Likewise, Jin et al. (2008) in fact found stCSDs in the cat visual cortex to generally be laterally more restricted than the monosynaptic arbor of the TC neurons, again pointing to a strongly localized stLFP signal in the lateral direction.

### Biophysical origin of local stLFP signal

The origin of these sharp decays of the LFP signals outside the synaptic target regions observed in Figure 7 can be understood on biophysical grounds by considering the underlying radial dependence of the CSD (Freeman and Nicholson, 1975; Pettersen et al., 2006; Pettersen and Einevoll, 2008) and the Poisson equation underlying the presently used volume-conductor theory.

**Figure 8.**
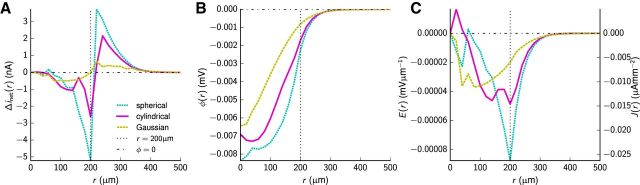
Biophysical origin of dependence of LFPs on lateral electrode position. ***A***, Radial current distribution Δ*i*_net_(*r*), i.e., total sum of transmembrane currents within spherical shells with thickness Δ*r* = 20 μm, as a function of each shell's outer radius (i.e., *r* = {20, 40, …, 500} μm). ***B***, LFP as a function of radius calculated by numerical evaluation of Equation 16 (and *C*_CSD_(*r*) corresponding to Δ*i*_net_ in ***D***). ***C***, Corresponding radial electrical field *E* = −*d*φ/*dr* and radial extracellular current density *J* = σ*E* as a function of radius. For numerical procedure for integration of Equations 16 and 17 underlying ***E*** and ***F***, see Materials and Methods.

Figure 8*A* shows the net transmembrane current, here denoted Δ*i*_net_, in spherical shells centered at the origin, as a function of outer shell radius *r*. This quantity was assessed by summing the total amount of transmembrane current within each spherical shell of thickness Δ*r*, making the corresponding CSD function *C*_CSD_(*r*) piecewise constant with radius *r* (compare Materials and Methods, Current-source density and potentials of concentric spherical shells). By assuming shells with thickness Δ*r* and constant current densities within each shell (i.e., no angular variation), Δ*i*_net_ is given as 4π*r*^2^(*C*_CSD_(*r*)Δ*r* at each shell's outer radius. In Figure 8*A* we observe that for small *r*, there is little net transmembrane current, i.e., the synaptic input currents providing sinks are approximately canceled by return currents providing sources. Closer to the edge at ∼200 μm, however, there is a net sink (i.e., Δ*i*_net_ < 0), as a larger fraction of neuronal membrane areas providing the return currents (sources) is located outside the target region. Outside the target region, the return currents will dominate and there will be a net source, i.e., Δ*i*_net_ changes sign and becomes positive. While this feature in the depicted spherically averaged CSD is, as expected, particularly prominent for the case with the spherical synaptic target region, it is also quite prominent for the cylindrical target-region case. With the smoother Gaussian target region, there is more sink-source cancellation resulting in only a weak net CSD sink–source pair.

These CSD profiles explain the key qualitative features of the observed decay of LFP. For the case of spherical symmetry, the Poisson equation ∇^2^φ = −*C*_CSD_/σ (Nunez and Srinivasan, 2006) can be evaluated numerically when *C*_CSD_(*r*) is known. The resulting potential φ(*r*), computed using *C*_CSD_(*r*) corresponding to the values for Δ*i*_net_ depicted in Figure 8*A*, is shown in Figure 8*B*. The resulting LFPs compare well with the exact numerical results along the *x*-axis in Figure 7*A*, demonstrating that qualitatively the radial dependence of the LFP is set up by a balanced set of spherical CSD shells straddling the boundary of the synaptic projection region.

The radial electric field *E* is given directly as −*d*φ/*dr*, and Figure 8*C* shows the electric field corresponding to the LFP depicted in Figure 8*B*. In volume-conductor theory, the extracellular current density *J* (current per area; Fig. 8*C*) equals this electrical field multiplied by the extracellular electric conductivity σ. Thus, the qualitative picture that emerges from Figure 8*C* is that the extracellular current flows inward from the return-current sources outside the synaptic projection region supplying the current absorbed by the synaptic current sinks in the center.

The strong “locality” of the LFP signatures (i.e., the sharp decay of the LFP signal outside the synaptic target region) is directly understood based on these paired sink–source shells at the boundary. In the hypothetical case with two infinitely thin balanced CSD shells (δ-shells) at the boundary, it follows from the Poisson equation that the LFP signal would be decaying “infinitely” sharply (i.e., be constant inside the synaptic target region, make an abrupt step at the boundary, and then be constant again outside the synaptic target region). As seen in Figure 8*A*, this situation is approximated most closely by the case with the spherical synaptic target region, and this is also the case where the sharpest decay of the LFP is observed at the boundary (Fig. 7*C*).

### Only iCSD methods can give reliable CSD estimates

The traditional way of analyzing multielectrode LFP data from layered brain structures, such as the cortex and hippocampus, has been by means of CSD analysis (Freeman and Nicholson, 1975; Pettersen et al., 2006). The CSD, corresponding to the net density of currents leaving or entering the extracellular medium, is by definition a more local measure of neural activity, and thus easier to interpret in terms of the underlying neural activity than the LFP signal itself. Further, the traditional way of CSD estimation from LFPs recorded by linear (laminar) multielectrodes has been by means of a “double spatial derivative” of the LFP signal recorded by equidistant electrodes spanning the cortical depth (Freeman and Nicholson, 1975; Mitzdorf, 1985). However, this analysis method does implicitly assume that neural activity (i.e., the CSD and the LFP) is constant in the lateral directions. Therefore, spurious current sinks and sources may be predicted if this assumption is violated, for example, due to a spatially restricted activation of the sensory cortex (Freeman and Nicholson, 1975; Pettersen et al., 2006). In contrast, with iCSD analysis, spatially restricted neural activation is straightforwardly handled by incorporation of the finite lateral size of the CSD directly into the CSD estimator (Pettersen et al., 2006).

As (1) the cortical LFP signature from a single thalamic afferent investigated here clearly represents a situation where the LFP is laterally restricted, and (2) we, unlike in the experimental situation, have direct access to the ground-truth CSD, we here investigated the accuracy of the CSD estimation methods within our model environment. Following the approach also pursued by Pettersen et al. (2008) and Łęski et al. (2011), we estimated CSDs from our model-based LFP data using both the tCSD and novel iCSD methods, and compared the estimates with ground-truth CSD data found directly by explicit summation of the transmembrane currents from all postsynaptic neurons in the populations.

**Figure 9.**
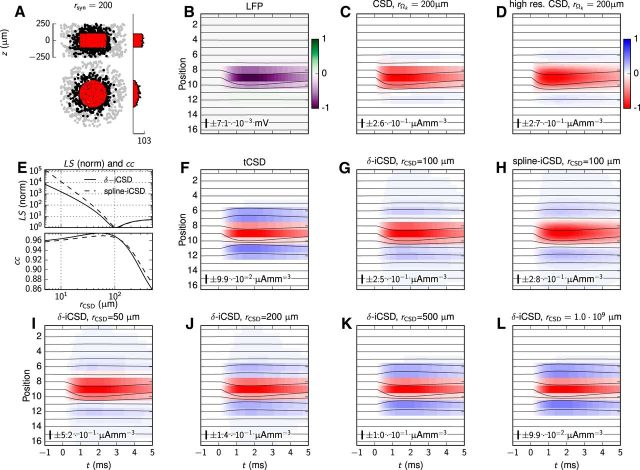
Comparison of ground-truth CSD and CSD estimates from model LFPs. The tCSD and iCSD methods were applied to model LFPs corresponding to an RS-cell population. The LFPs resulted from a synapse distribution within a cylinder with *r*_syn_ = *h*_syn_ = 200 μm, and zero offset μ*_z_*. Synapses were activated at *t* = 0 ms, and other parameters were similar to the default parameters (Table 1). The projection resulted in 666 postsynaptic neurons and 1758 dendritic synapses. ***A***, Illustration of the postsynaptic RS-cell population and synapse positions. The scatter plots show the synaptic distributions projected in the *xz* and *xy* planes, respectively, with corresponding histograms of synaptic locations. Gray dots denote somas of neurons without synapses; black dots denote postsynaptic neurons; red dots denote the synapse locations. The histograms show the spatial distribution of synapse locations along the *x*-axis and *z*-axis, respectively. The single tick label denotes the maximum synapse count within 10 μm bins along the depicted axes. ***B***, LFP response from TC input. ***C***, Ground-truth CSD, filtered. Spatial resolution corresponded to the LFP recording, i.e., *h_z_* = 100 μm. The ground-truth CSDs correspond to a radius *r*_Ω_*k*__ = *r*_syn_ = 200 μm. ***D***, Ground-truth CSDs calculated with increased spatial resolution (*h_z_* = 20 μm), filtered. The ground-truth CSDs correspond to a radius *r*_Ω_*k*__ = *r*_syn_ = 200 μm. ***E***, Deviations between ground-truth CSD (***C and D***) and δ-iCSD and spline-iCSD estimates as a function of source radius *r*_CSD_. The least-square error measure *LS* is defined in Equation 13, and values are normalized such that the global minimum is 1 (occurring for the value of *r*_CSD_ giving the best fit). Also shown is the correlation coefficient, i.e., Pearson product–moment correlation coefficient, *cc*, between ground-truth CSD and the iCSD estimates. ***F***, Standard CSD estimate from model LFP in ***B***. ***G***, δ-iCSD method estimate based on model LFP in ***B***, *r*_CSD_ = 100 μm. ***H***, Spline-iCSD method estimate based on model LFP in ***B***, *r*_CSD_ = 100 μm. ***I–L***, δ-iCSD estimates for a set of different values for *r*_CSD_, i.e., 50, 200, 500 and 10^9^ μm, respectively. The number and scale bar inside ***B–D*** and ***F–L*** describe the amplitude of the line plots. The number also gives the LFP or CSD values corresponding to ±1 of the color bar.

In Figure 9 we show the results for an example with the RS-cell population with cylindrical distribution of synaptic inputs with *r*_syn_ = *h*_syn_ = 200 μm giving the LFP signature, as recorded by a centered laminar electrode, depicted in Figure 9*B*. The ground-truth CSDs (Fig. 9*C*,*D*) were calculated using a lateral “averaging” radius *r*_Ω_*k*__ set equal to *r*_syn_ (see Materials and Methods, Ground-truth CSD), and with two different spatial resolutions (*h*_Ω_*k*__ = 100 μm in Fig. 9*C*; *h*_Ω_*k*__ = 20 μm in Fig. 9*D*) for easier comparison with the various CSD analysis methods.

The CSD estimate found from using the tCSD method (including the method of Vaknin for outermost contacts; see Materials and Methods) on the LFP data in Figure 9*B* is shown in Figure 9*F*. By comparing this with the corresponding ground-truth CSD in Figure 9*C*, we observe several substantial estimation errors: (1) large spurious current sources are predicted above and below the true current sink, (2) the central sink is predicted to be too narrow, and (3) the CSD amplitude is underestimated by approximately a factor of three. These errors are not surprising given that the tCSD method implicitly assumes that the CSD activation patterns have infinite lateral extents, while activation in reality extends only a few hundred micrometers away from the recording electrode (Pettersen et al., 2006, 2008; compare Fig. 7*B*). In the present situation, the lateral extent of the CSD sources was so small that in fact the LFP itself (Fig. 9*B*) was a better predictor of the shape of the CSD than the tCSD estimate involving a double spatial derivative of the LFP (Pettersen et al., 2006).

Next, we investigated CSD estimates by means of iCSD methods explicitly assuming laterally confined CSDs. Unlike for the tCSD method, we here had to specify the assumed radius *r*_CSD_ of the underlying columnar CSD distribution. In Figure 9 we show the resulting CSD estimates found from the δ-iCSD method assuming four different values for the columnar radius [i.e., *r*_CSD_ = 50 μm (Fig. 9*I*), *r*_CSD_ = 100 μm (Fig. 9*G*), *r*_CSD_ = 200 μm (Fig. 9*J*), *r*_CSD_ = 500 μm (Fig. 9*K*), and *r*_CSD_ = 10^9^ μm effectively corresponding to *r*_CSD_ → ∞ (Fig. 9*L*)]. A visual comparison with the ground-truth result (Fig. 9*C*) suggests that a good agreement between estimated and ground-truth CSD was obtained for *r*_CSD_ = 100 μm (Fig. 9*G*). The iCSD estimates for *r*_CSD_ = 200 μm on the other hand overestimated the amplitude source above and below the central current sink, and the iCSD estimates for *r*_CSD_ = 500 μm even more so. In fact, the iCSD estimate for *r*_CSD_ = 500 μm resembles the estimate from using the traditional “double spatial derivative” (tCSD) method depicted in Figure 9*F*. This reflects that in the limit *r*_CSD_ → ∞ the δ-iCSD becomes identical to the tCSD method (Pettersen et al., 2006), here exemplified by comparison of Figure 9*F* and Figure 9*L*. The δ-iCSD estimate for *r*_CSD_ = 50 μm (Fig. 9*I*) has a similar shape as the ground-truth CSD (Fig. 9*C*), but the amplitude was here overestimated by approximately a factor of two.

The detailed dependence of CSD estimation error [i.e., the *LS* values (Eq. 13) normalized by the minimum value for all source radii *r*_CSD_] is shown in Figure 9*E*. Here we observe that the smallest error for the δ-iCSD method was obtained for *r*_CSD_ = 100 μm, more precisely for *r*_CSD_ = 105 μm. This error measure penalizes incorrect overall magnitude deviations in the estimated CSD. However, often an accurate estimate of the shape of the CSD profile (i.e., a high correlation between the estimate and the ground truth) is considered more important. In Figure 9*E*, we thus also plot the pairwise correlation coefficient (*cc*) as a function of *r*_CSD_. Here the optimal value of *r*_CSD_ is found to be only 55 μm, in accordance with the above visual observation of close overlap with the ground-truth shape for the δ-iCSD estimate for *r*_CSD_ = 50 μm.

The estimation error for the spline-iCSD method when comparing with the corresponding “high-resolution” ground-truth CSD (Fig. 9*D*) shows a similar dependence on *r*_CSD_ (Fig. 9 *E*). Specifically, we found that the best spline-iCSD estimate was obtained for *r*_CSD_ = 110 μm. The spline-iCSD estimate for *r*_CSD_ = 100 μm is shown in Figure 9*H*. Also for the spline-iCSD method the correlation coefficient was found to have its maximum for a much smaller value of *r*_CSD_ (i.e., *r*_CSD_ = 60 μm), compared with the value giving the smallest error.

Notably, the value of the columnar radius giving the smallest iCSD estimation error (i.e., *r*_CSD_ = ∼100 μm) is approximately half of the radius of the synaptic target region (*r*_syn_ = 200 μm). This indicates that the presently used iCSD method assumes a monophasic profile of the underlying CSD in the lateral directions, while the real, ground-truth CSDs largely have biphasic lateral profiles with a current source close to the center surrounded by more peripheral current sources. As a result, the lowest error of a single monophasic lateral CSD profile turned out to be approximately half of the synaptic target region, but note that this fraction may in general depend on the choice of radius used to compute the ground-truth CSD. Also, such biphasic lateral profiles could in principle be assumed in similar iCSD estimations, but this was not pursued here.

### CSD analysis of experimental stLFPs

**Figure 10.**
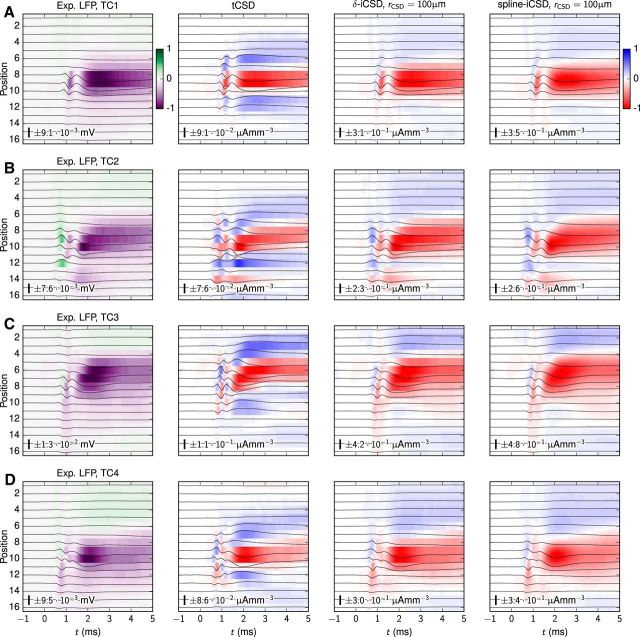
CSD estimates from experimental LFPs. ***A***, Experimental postsynaptic LFPs (column 1), corresponding to Swadlow et al., (2002, their Fig. 1*B–N1*), with corresponding standard CSD (column 2), δ-iCSD (column 3), and spline-iCSD estimates (column 4). ***B***, Experimental postsynaptic LFP and CSD estimates (Swadlow et al., 2002, their Fig. 1*B–N2*). ***C***, Experimental postsynaptic LFP and CSD estimates (Stoelzel et al., 2008, their Fig. 1*E*). ***D***, Experimental postsynaptic LFP and CSD estimates (Stoelzel et al., 2008, their Fig. 2*A1*). For the iCSD methods, a column radius of *r*_CSD_ = 100 μm was assumed. In each panel, the number and scale bar describe the amplitude of the line plots. The number also gives the LFP/CSD values corresponding to ±1 of the color bar.

With iCSD estimation methods tuned to give an excellent agreement with model ground-truth data, i.e., by assuming a source radius *r*_CSD_ = 100 μm, we now return to the experimental recordings of monosynaptic LFP signatures shown earlier in the top row of Figure 4. The resulting CSD estimates are shown in Figure 10 where the first two rows (Fig. 10*A*, TC1; Fig. 10*B*, TC2) correspond to the monosynaptic LFP recordings from the rabbit somatosensory (S1) cortex (Swadlow et al., 2002), while the last two rows (Fig. 10*C*, TC3; Fig. 10*D*, TC4) correspond to the recordings from rabbit visual (V1) cortex (Stoelzel et al., 2008). The panels in the first column correspond to the experimental LFPs, the second column to CSD estimates from standard CSD (tCSD) analysis, the third to CSD estimates from the above optimized δ-iCSD method, and the fourth to CSD estimates from the correspondingly optimized spline-iCSD method.

Compared with the tCSD method, the iCSD methods predicted only small current sources, predominantly above the dominant current sink. This vertical asymmetry may reflect that the upper current sources are due to return currents if some of the target dendrites in layer 4 are basal dendrites of pyramidal cells or so-called star-pyramid cells (Oberlaender et al., 2012), i.e., cells with a stellate-like bush in layer 4 accompanied by an apical stalk stretching into the superficial layers. Our results may hint that this effect is more prominent in V1 (TC3, TC4) than in S1 (TC1, TC2). If so, one possible explanation is that such pyramidal cells may be more prolific in V1 than S1. For example, in the cat visual cortex, approximately one-third of excitatory cells in layer 4 have been suggested to be of this type (Binzegger et al., 2004). In the rat barrel cortex and rat hind-limb somatosensory cortex, layer-4 excitatory cells comprise a mix of pyramidal cells, star pyramidal cells, and spiny stellate cells (Oberlaender et al., 2012; Markram et al., 2015). Stellate cells do not occur in layer 4 of the rat primary visual cortex (Peters and Kara, 1985; Iurilli et al., 2012; Harris and Shepherd, 2015). Also, layer 4 of the mouse visual cortex appears to comprise a large fraction of pyramidal cells (Allen Brain Atlas, http://celltypes.brain-map.org; RRID:SRC_014806).

For the response corresponding to cell TC2, the CSD signature exposed an additional postsynaptic sink in deeper cortical lamina. This smaller current sink, starting earlier than the dominant sink above, presumably reflects synaptic activation of deep layer-5 or layer-6 cells from the incoming action potential along the afferent thalamic axon (Swadlow et al., 2002). Unlike TC1, the particular signature of TC2 also exhibited a faster onset of the CSD signal at the lower edge of the central CSD sink. This may be caused by preferential targeting of the FS-cell population slightly deeper in the cortex compared with the RS-cell population.

## Discussion

In the present paper, we have investigated the synaptic LFP footprint of individual TC neurons activating cells in layer 4 of the sensory cortex. The modeling study was motivated by a set of experimental studies (Swadlow et al., 2002; Jin et al., 2008, 2011; Stoelzel et al., 2008, 2009) where simultaneous recording of spikes of TC neurons and cortical LFPs across lamina allowed for the estimation of stLFPs and associated CSDs directly reflecting this monosynaptic connection. To take full advantage of such measurements, it is crucial to fully understand the link between the stLFP signal that is measured and the underlying synaptic action. This in turn requires biophysical modeling of the type done here (Einevoll et al., 2013).

Our model for the postsynaptic target populations comprised 5000 morphologically reconstructed cells of which 80% were RS neurons with a spiny stellate-cell morphology and 20% FS neurons with a large basket-cell morphology. The model cells were passive, and the parameters describing their electrical properties and their thalamic synaptic input currents were tuned to give intracellular responses to synaptic activation (EPSPs, EPSCs) in accordance with the experimental literature (Figs. 2, 3). The extracellular potential, here referred to as LFP, was calculated based on computed transmembrane currents in the postsynaptic neurons using a well established biophysical modeling scheme from volume-conductor theory (Rall and Shepherd, 1968; Holt and Koch, 1999; Lindén et al., 2013).

A first observation was that the model could accurately reproduce previously recorded experimental stLFPs (Swadlow et al., 2002; Stoelzel et al., 2008), both in terms of overall shape and amplitude, when contributions from the RS-cell and FS-cell populations were added (Fig. 4). Our modeling suggests, in particular, that the rapid onset of the stLFP is provided by contributions from the FS cells, while the RS cells contributed most of the latter part of the stLFP signature. An a priori unknown is the spatial extent of the synaptic projection pattern of the TC neuron setting up the stLFPs, but we found that this model parameter had little effect on the shape of the spatiotemporal stLFP profile (Fig. 5). However, the stLFP amplitude was observed to be about a factor two larger for the largest synaptic projection pattern (cylindrical projection with radius 400 μm) compared with the smallest size considered (radius 50 μm). Note that our considered range is in accordance with recent findings from reconstructed VPM axons and bouton densities in layer 4 of rat barrel columns, where the lateral extent was found to be 200–400 μm (Oberlaender et al., 2012).

A key question for interpretation of experiments regards the locality of the stLFP signature: how does the recorded stLFP depend on the lateral position of the electrode in the cortex compared with the position of the synaptic projection pattern of the thalamic neuron? We found that while the stLFP signal is largest when the electrode is placed in the center of the synaptic projection, the signal decays little when the electrode is moved laterally as long as it remains within the synaptic target zone (Fig. 6). However, it dropped sharply when the recording electrode was placed outside this zone, in particular for the spherical and cylindrical distributions with sharp boundaries (Fig. 6). This sharp drop could be understood from effective cancellation of CSDs close to the center, accompanied by a balanced sink–source pair of CSD shells at the synaptic target-region boundary. This characteristic CSD pattern is due to the nonpyramidal morphologies of the target cells implying a “closed-field” arrangement of the LFP sources (Lorente de No, 1947; Lindén et al., 2010), not only for the spherically symmetric situations (Fig. 8, spherical, Gaussian), but also for the cylindrical target-pattern case.

A qualitatively similar sharp drop in LFP amplitude was observed outside a synaptically activated population of cortical pyramidal neurons (Lindén et al., 2011; Łęski et al., 2013). Here the set-up was slightly different however: an “open-field” arrangement (Lorente de No, 1947; Lindén et al., 2010) where the somas of the postsynaptic target population (rather than afferent synapses) were assumed to be uniformly distributed on a cylindrical disc.

The standard method for analyzing LFP recordings with linear multielectrodes has been to do a CSD analysis (Pitts, 1952; Nicholson and Freeman, 1975; Mitzdorf, 1985) with the CSD estimated from a discrete spatial double-derivate of the LFP in the depth direction. This inherently assumes that the neural activity does not vary in the lateral directions (Nicholson and Freeman, 1975). For the LFP generated by a focal activation of cortex by a single TC afferent like here, this condition cannot be expected to be fulfilled. In contrast, the iCSD method allows for reliable CSD estimation also with localized neural activity (Pettersen et al., 2006; Łęski et al., 2007, 2011). With access to the ground-truth CSD in our model system, we could objectively evaluate the various candidate CSD estimators. For the present application, we found that both the δ-iCSD and spline-iCSD methods (Pettersen et al., 2006) provide accurate estimates for the CSD depth profile when appropriate values for cylindrical source parameter *r*_CSD_ are chosen in the CSD estimator. For a cylindrical synaptic target of radius 200 μm, the best CSD estimates in terms of deviation error and shape were obtained for *r*_CSD_ ≈ 100 μm and *r*_CSD_ ≈ 60 μm, respectively (Fig. 9). In contrast, the traditional CSD method using the double spatial derivative gave poor predictions for the CSD profile and predicted, for example, far too large current sources above and below the central current sink (Fig. 9). Thus, we conclude that CSD estimation for focal stLFPs should be performed using the iCSD method, or other new CSD estimation methods based on inverting the electrostatic forward solution, such as the kernel CSD method (Potworowski et al., 2012) and others (Kropf and Shmuel, 2016).

Our present model investigation is “chimeric” as it uses neuronal morphologies reconstructed from the rat somatosensory cortex (Wang et al., 2002), combined with passive neuron parameters and synaptic currents tuned to intracellular recordings from the rat and mouse (Beierlein et al., 2003; Hull et al., 2009), to predict stLFP signatures that were compared with experimental results from rabbit cortices (Swadlow et al., 2002; Stoelzel et al., 2008). While an analog single-species study should be pursued when such data are available, we expect the gross features of the stLFPs would be largely unaffected. With the recent release of the Neocortical Microcircuit Collaboration Portal (http://bbp.epfl.ch/nmc-portal), numerous multicompartmental layer-4 model neurons from the rat somatosensory cortex have become available (Markram et al., 2015; Ramaswamy et al., 2015), opening the way for a systematic investigation of the effect of varying the neuronal properties of the layer-4 cells postsynaptic to the thalamic afferents.

We assumed that each thalamic afferent does not form synapses on every possible target neuron in cortical layer 4, allowing us to reduce the cell densities in our model populations. Our model included 5000 cells (4000 RS cells, 1000 FS cells), which gives an overall density of target layer-4 neurons of ∼13,000 per mm^−3^, which is ∼10× smaller than the total layer-4 neuron density estimated in the rat somatosensory cortex by Meyer et al. (2010). This model choice gave stLFP amplitudes similar to experimental observations in rabbit somatosensory and visual cortices (Swadlow et al., 2002; Stoelzel et al., 2008), but note that also a model with a lower fraction of FS cells (Meyer et al., 2011) was found to be accordance with these experiments (Fig. 4).

Unlike in recurrent cortical networks where both excitatory and inhibitory inputs drive the LFP (Haider et al., 2016; Teleńczuk et al., 2017), only excitatory inputs from TC neurons contribute here. Further, the experimental stLFPs targeted by the modeling were computed by spike-trigger averaging on spontaneous spikes in the TC neurons, which in general did not drive the postsynaptic cells to fire action potentials (Swadlow et al., 2002). Thus the experimental stLFPs did expectedly not contain sizable contributions from the suprathreshold ion-channel currents underlying spiking. However, subthreshold active dendritic conductances, such as the *I*_h_ current, can affect LFP signatures of synaptic activation, but likely only for signal frequencies below a few 10s of hertz (Ness et al., 2016). For the rapid (i.e., high-frequency) stLFP signals considered here, lasting <10 ms, we thus expect small effects from active dendritic conductances.

In the present study we assumed an infinite, homogeneous (same everywhere), isotropic (same in all directions) and frequency-independent extracellular conductivity σ. Within cortical gray matter there seems to be negligible variation of σ between cortical layers (Goto et al., 2010), and for the present setup there should be negligible effects from steps in extracellular conductivity at the top of the cortex and at the white-matter boundary (Pettersen et al., 2006). Goto et al. (2010) observed, however, ≤50% larger σ in the depth direction compared with the lateral directions. If warranted, such anisotropy can easily be incorporated in the present forward-modeling scheme (Pettersen et al., 2012) and would modify the predicted stLFP profiles somewhat (Ness et al., 2015, their Fig. 6), but we would not expect such anisotropy to change any of our conclusions. Recent experiments have only found a small frequency dependence of the extracellular conductivity σ at relevant electrophysiological frequencies (*f* ≲ 1000 Hz; Logothetis et al., 2007; Elbohouty, 2013; Wagner et al., 2014; Miceli et al., 2017; but see Gabriel et al., 1996; Bédard and Destexhe, 2009). In any case, the present scheme could still be applied with frequency-dependent conductivity by means of Fourier decomposition of the stLFP (cf. Ness et al., 2015).

Several large-scale modeling projects aimed at biophysically detailed simulation of cortical networks are on the way where also population signals like the LFP are to be computed (Kandel et al., 2013). In this context, the present study may be seen as a pilot study since the stLFP represents an unusually well controlled population signal. The success of the present models in predicting experimental stLFP signatures based on previous single-neuron recordings is thus encouraging.
